# Preclinical Evaluation of a ^177^Lu-Labeled Gastrin-Releasing Peptide Receptor Antagonist and Prostate Cancer Treatment with Monotherapy and in Combination with Everolimus

**DOI:** 10.1021/acsptsci.5c00491

**Published:** 2025-10-28

**Authors:** Naveen Kumar, Adrianna Bilinska, Elena Menéndez, Tilman Läppchen, Panagiotis Kanellopoulos, Anna Orlova, Frank Rösch, Axel Rominger, Eleni Gourni

**Affiliations:** 1 Department of Nuclear Medicine, Inselspital, Bern University Hospital, University of Bern, Bern 3010, Switzerland; 2 Graduate School of Cellular and Biomedical Sciences, University of Bern, Bern 3010, Switzerland; 3 Department of Medicinal Chemistry, Uppsala University, Uppsala 751 83, Sweden; 4 Department of Chemistry—TRIGA site, Johannes Gutenberg—University of Mainz, Mainz 55128, Germany

**Keywords:** radiolabeled GRPR antagonists, mTOR inhibitor, everolimus, fractionated therapeutic dose, monotherapy, combination

## Abstract

This study evaluates the potential of a ^177^Lu-labeled GRPR-targeting antagonist as a radiotherapeutic agent for tumors expressing the gastrin-releasing peptide receptor (GRPR). The therapeutic effect of the radioligand was investigated both as a monotherapy and in combination with the mTOR inhibitor everolimus. The GRPR antagonist, LF1 (AAZTA^5^-Pip-d-Phe-Gln-Trp-Ala-Val-Gly-His-Sta-Leu-NH_2_), was synthesized using the chelator AAZTA^5^ linked via a 4-amino-1-carboxymethylpiperidine (Pip) spacer and radiolabeled with lutetium-177. The preclinical evaluation included assessments of binding kinetics, blood and organ clearance, plasma protein binding, and metabolic stability. SPECT/CT imaging and biodistribution studies were performed in mice bearing PC3 xenograft tumors. To assess its therapeutic efficacy, PC-3-mice were treated with [^177^Lu]­Lu-LF1 either alone or following everolimus pretreatment. [^177^Lu]­Lu-LF1 showed high binding affinity (K_d_ = 0.12 ± 0.01 nM) and favorable pharmacokinetics, including rapid blood clearance and low plasma protein binding (2–3% at 5 and 15 min p.i.). Although subject to enzymatic degradation, the radioligand demonstrated high, sustained, and specific tumor uptake (42 ± 5.0% IA/g at 1 h and 3.9 ± 1.1% IA/g at 144 h p.i.). Pancreatic uptake cleared quickly, allowing for high-contrast SPECT/CT imaging. Therapeutically, tumors treated with 93 MBq of [^177^Lu]­Lu-LF1 grew more slowly than those treated with 41 MBq. The combination of everolimus and [^177^Lu]­Lu-LF1 resulted in significant tumor growth inhibition, compared to the relevant monotherapies with either [^177^Lu]­Lu-LF1 or everolimus. [^177^Lu]­Lu-LF1 shows promise as a therapeutic radioligand for GRPR-positive prostate cancer, offering high tumor uptake and rapid clearance from nontarget tissues. Mice bearing PC3 xenograft tumors were well tolerated and demonstrated enhanced therapeutic efficacy when combined with everolimus.

## Graphical abstract

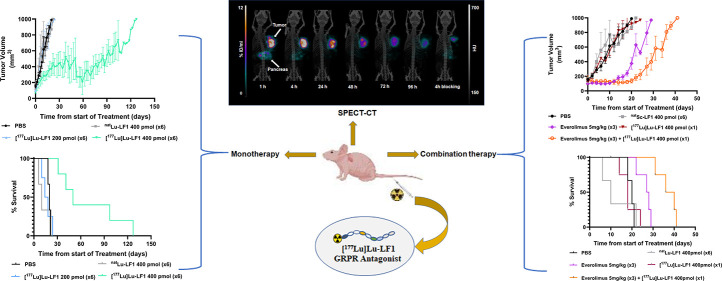

## Introduction

The gastrin-releasing peptide receptor (GRPR) plays a critical role in numerous physiological and pathological processes including the progression of various cancers. GRPR is significantly overexpressed in several tumor types, including prostate, breast, pancreatic, and small-cell lung cancers, where it promotes cell proliferation, angiogenesis, and metastasis through activation of signaling pathways such as MAPK and PI3K/AKT.^1,2^ This elevated GRPR expression in tumors, has positioned it as a compelling target for both cancer imaging and therapy, particularly in the context of nuclear medicine. A broad spectrum of GRPR-targeted peptidic analogues, encompassing both agonistic and antagonistic profiles, have been developed and extensively evaluated, demonstrating significant potential. However, early clinical attempts utilizing GRPR-targeting agonists were unsuccessful, primarily due to significant gastrointestinal side effects observed at therapeutic peptide doses.^3^ To address these limitations, GRPR antagonists were promptly introduced and evaluated for their potential application for both imaging and radionuclide therapy.^4,5^ The transition from agonists to antagonists was further supported by findings from somatostatin receptor research, which revealed that radiolabeled antagonists can bind multiple receptor sites and exhibit superior pharmacokinetic profiles compared to agonists.^6^ These insights have further supported the clinical interest in GRPR-targeting antagonists as a safer and potentially more effective strategy for theranostic purposes.

In the evolving landscape of prostate cancer imaging, the complementary use of GRPR and prostate-specific membrane antigen (PSMA) radioligands marks a significant step forward. By combining these two approaches, clinicians can achieve more accurate diagnosis, better staging, and improved therapeutic guidance, particularly for challenging cases such as PSMA-negative or heterogeneous tumors.^7^ Beyond diagnostic applications, the emergence of new-generation GRPR antagonist-based radiopeptides is paving the way for realistic theranostic approaches, broadening the scope of precision medicine in prostate cancer management.

The application of radiolabeled GRPR-targeting agents such as RM2, AMTG, SAR-BBN (NCT05633160) and NeoB (NCT03872778) as theranostic tools has demonstrated significant efficacy in the diagnosis and treatment of various cancers that exhibit high expression of the GRPR. These malignancies include prostate cancer, breast cancer, gastrointestinal tumors, lung cancer, and glioblastomas, as supported by multiple clinical studies and trials.^4,8–13^ By combining diagnostic imaging and targeted radionuclide therapy, they have shown promising results in enhancing tumor detection and delivering selective cytotoxic radiation to cancerous tissues.

Despite these encouraging developments, the clinical translation of GRPR-targeting radioligands faces several key challenges. One of the primary limitations is their susceptibility to enzymatic degradation in vivo, which significantly shortens their biological half-life and limits their therapeutic efficacy.^13,14^ This rapid metabolic breakdown necessitates ongoing efforts to improve the chemical stability, receptor-binding affinity, and pharmacokinetic profiles of these peptide-based radiopharmaceuticals.^13–19^

This study provides a detailed preclinical assessment of LF1 (AAZTA^5^-Pip-d-Phe-Gln-Trp-Ala-Val-Gly-His-Sta-Leu-NH_2_), a GRPR-targeting statine based antagonist developed by our group.^20^ LF1 is functionalized with the chelator AAZTA^5^ (6-[Bis­(carboxymethyl)­amino]-1,4-bis­(carboxymethyl)-6-methyl-1,4-diazepane) via the positely charged Pip spacer (4-amino-1-carboxymethylpiperidine) and was previously radiolabeled with a variety of radionuclides and characterized in vitro by us.^20^ Here, we further assess [^177^Lu]­Lu-LF1 through in vitro and in vivo studies, including GRPR-binding assays, pharmacokinetics, metabolic stability, biodistribution, and SPECT/CT imaging. Its therapeutic efficacy was tested in prostate cancer models using two regimens: [^177^Lu]­Lu-LF1 alone and combined with the mTOR inhibitor everolimus, aiming to enhance treatment response through the mTOR pathway inhibition.^21^

## Results and Discussion

### Radiolabeling, Quality Control, and Stability of [^177^Lu]­Lu-LF1

The favorable properties of AAZTA^5^ enabled fast and efficient synthesis of [^177^Lu]­Lu-LF1 under mild conditions. The molar activities ranged from 10 to 44 GBq/μmol, depending on the experiment. The radiochemical yield and purity exceeded 99%, confirmed by radio-TLC and radio-HPLC. The tracer remained highly stable (99 ± 0.01%, *n* = 10) up to 72 h postlabeling. After 6 days, slight tailing was observed, and the radiochemical stability was assessed as 88% after integration of the signal (Figures S1 and S2, in the Supporting Information).

The mesocyclic structure of AAZTA^5^, when conjugated to a GRPR antagonist, enables efficient radiolabeling under mild conditions with high molar activities, up to 44 GBq/μmol without further purification, suggesting even higher values are possible. However, previous work from our group^20^ indicated that AAZTA^5^ is suboptimal for gallium-68, limiting its use in [^177^Lu]­Lu-LF1/[^68^Ga]­Ga-LF1 theranostic pairs. This challenge can be overcome by employing RM2, a GRPR-targeted tracer effectively labeled with gallium-68.^22^ Despite differences in chelators (DOTA vs AAZTA^5^), RM2 and LF1 share similar peptide and spacer structure, supporting their potential as a complementary theranostic pair. Moreover, AAZTA^5^ shows promise for other radionuclide pairs such as scandium-44/47 and copper-64/67,^23^ warranting further investigation. The longer half-lives of scandium-44 (3.97 h) and copper-64 (12.7 h) compared to gallium-68 (67.7 min) allow for delayed imaging, which may enhance GRPR-targeted imaging due to improved background clearance, as shown in both preclinical^24,25^ and clinical studies.^26^

### Binding Affinity and Kinetics

[^177^Lu]­Lu-LF1 exhibited a high binding affinity with a K_d_ value of 0.12 ± 0.01 nM, indicating strong and specific receptor interaction. The kinetic analysis revealed a moderate association rate constant (K_on_) of 2.5 × 10^5^ ± 0.2 × 10^5^ M^–^^1^s^–^^1^ and a very slow dissociation rate constant (K_off_) of 3.2 × 10^–5^ ± 0.1 × 10^–5^ s^–1^ (Figure 1). [^177^Lu]­Lu-LF1 showed an improved kinetic profile compared to the existing GRPR antagonist [^177^Lu]­Lu-RM2 (K_d_ = 5.4 ± 0.8 nM and IC_50_ = 7.7 ± 3.3 nM).^20,27^ The slow off-rate in [^177^Lu]­Lu-LF1 is particularly favorable for therapeutic applications, as it suggests prolonged receptor occupancy and sustained radiation delivery to GRPR-expressing tumors. These results highlight the strong potential of [^177^Lu]­Lu-LF1 as a GRPR-targeted radiopharmaceutical, combining both high receptor affinity and favorable kinetic for effective tumor targeting and retention. The much lower K_d_ obtained with LigandTracer compared to previously reported IC_50_ values for RM2 ^18,27^ and K_d_ value of LF1 ^20^ might appear to indicate a major increase in affinity, but this discrepancy should be interpreted with caution. The values were generated using different experimental strategies, cell-based competition assays versus real-time kinetic binding, which are influenced by factors such as tracer concentration, incubation conditions, receptor density, and data-fitting models. As such, the LigandTracer-derived K_d_ of 0.1 nM should not be directly compared with IC_50_ values from earlier assays, but rather considered a complementary measure that provides higher-resolution insight into binding kinetics.

**Figure 1:**
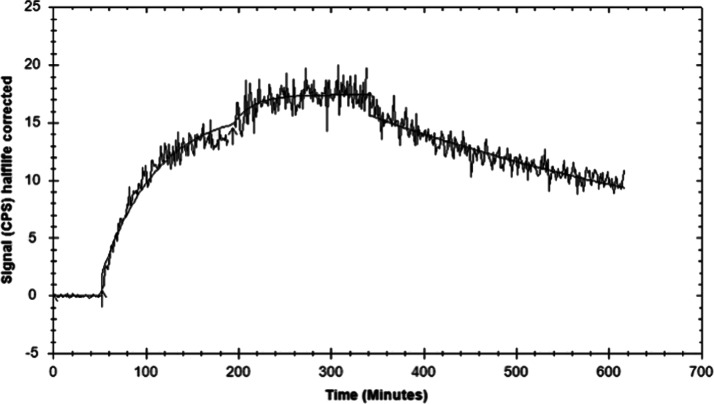
Sensogram of the kinetic profile of [^177^Lu]­Lu-LF1 in PC-3 cells using LigantTracer Gray. For the association phase measurements, two increasing concentration were used, 1 and 3 nM of [^177^Lu]­Lu-LF1 in complete medium.

### Biodistribution Studies

The biodistribution and tumor-to-tissue ratios of [^177^Lu]­Lu-LF1 are summarized in Figure 2 and Table 1, with detailed ex vivo data provided in Table S1 (supplementary). [^177^Lu]­Lu-LF1 showed rapid blood clearance, with ∼ 1% IA/g at 1 h p.i., further decreasing over time. Tumor uptake was high and rapid (>40% IA/g at 1 and 4 h p.i.), with slow clearance, retaining 3.9 ± 1.1% IA/g at 144 h p.i. High uptake was initially observed in GRPR-rich organs such as the pancreas (71 ± 8.1% IA/g at 1 h p.i.), however, decreased quickly, dropping by a factor of 4.7 at 4 h and to <1% IA/g at later time points. This finding is in agreement with the behavior previously observed with other radiolabeled GRPR antagonists.^18,24,25,28^ Tumor-to-pancreas ratios rose from 0.5 at 1 h to >20 at later time points. Renal excretion was the primary elimination route, with low liver uptake (<1% IA/g) across the investigated time points. Kidney uptake peaked at 7.1 ± 1.1% IA/g at 1 h p.i., dropping below 2% IA/g by 24 h. GRPR-specific targeting was confirmed by a 70% and 95% reduction in tumor and pancreas uptake, respectively, following coinjection with the blocking agent.

**Figure 2:**
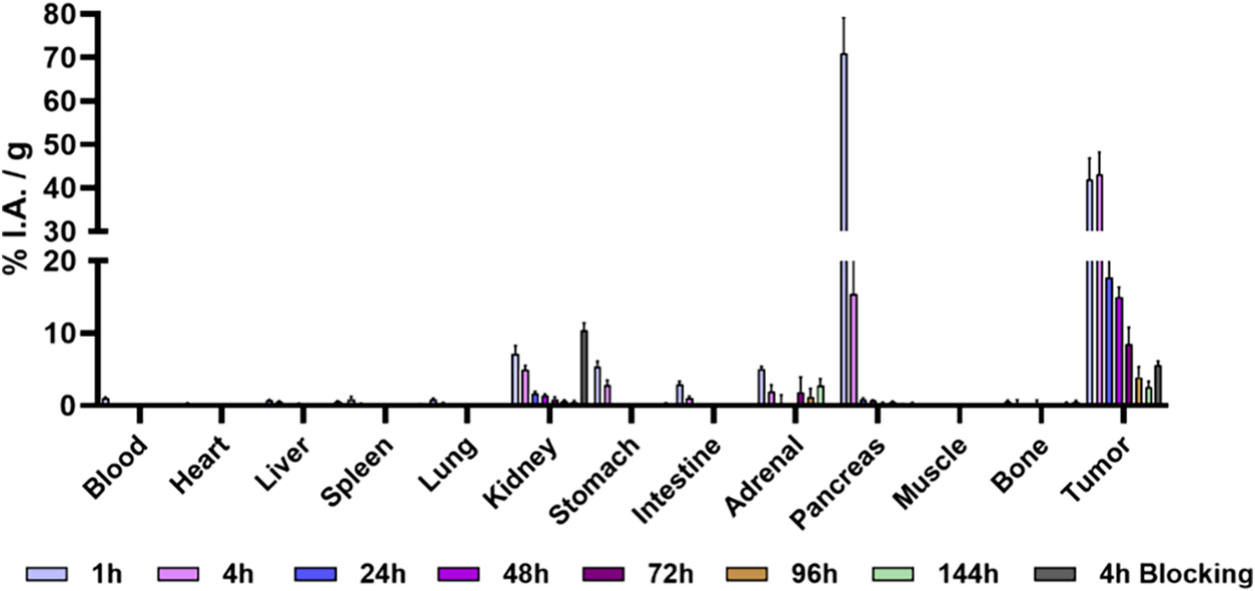
Biodistribution data of [^177^Lu]­Lu-LF1 in PC-3-mice at 1, 4, 24, 48, 72, 96, and 144 h p.i along with blocking studies data at 4 h p.i. Data have been calculated as %IA/g of tissue and are presented as mean ± SD (*n* = 3–4).

**Table 1:** Tumor to Organ (Blood, Liver, Kidney, Muscle, and Pancreas) Ratios of [^177^Lu]­Lu-LF1 in PC-3-mice at 1, 4, 24, 48, 72, 96, and 144 h p.i

	1 h	4 h	24 h	48 h	72 h	96 h	144 h
tumor/blood	40 ± 1.2	272 ± 35	>500	>500	>500	>500	>500
tumor/liver	57 ± 7.2	76 ± 8.1	72 ± 9.8	58 ± 7.3	39 ± 4.1	25 ± 1.3	13 ± 2.5
tumor/kidney	5.9 ± 0.3	8.8 ± 0.8	10 ± 1.0	11 ± 1.8	9.9 ± 1.8	6.1 ± 0.0	4.9 ± 0.2
tumor/muscle	271 ± 40	251 ± 39	210 ± 24	234 ± 77	121 ± 60	336 ± 230	48.7 ± 0
tumor/pancreas	0.59 ± 0.0	3.1 ± 0.9	21 ± 4.7	20 ± 2.0	25 ± 3.9	8.6 ± 1	9.0 ± 3.1

Among the evaluated GRPR-based radioligands (RM2, RM26, AMTG, NeoB, RGD-Glu-[DO3A]-6-Ahx-RM2) (Table S2), [^177^Lu]­Lu-LF1 exhibited the highest tumor uptake (42%IA/g at 1 h p.i. and 18%IA/g at 24 h p.i.) demonstrating strong and sustained tumor targeting.^18,24,28,29^ When comparing these results, it is important to account for differences in experimental conditions between the studies, particularly with respect to the mice strain and gender. [^177^Lu]­Lu-LF1 showed significantly high pancreatic uptake at 1 h p.i. (70%IA/g), which declined sharply to 0.9%IA/g by 24 h p.i., indicating rapid clearance from GRPR-rich healthy tissues. Additionally, kidney and liver uptake remained consistently low, reflecting a favorable pharmacokinetic profile. In comparison, [^177^Lu]­Lu-NeoB displayed substantially lower tumor uptake (9%IA/g at 1 h p.i. and 8%IA/g at 24 h p.i.), but with a longer biological half-life (*t*_1/2_ = 50 h), potentially advantageous for delayed imaging or therapeutic applications.^24^ However, its pancreatic retention at 24 h p.i. (4%IA/g) suggests slower clearance from nontarget tissues, which may raise concerns regarding off-target exposure.^24^ The AMTG analogs labeled with lutetium-177 and terbium-161, particularly [^161^Tb]­Tb-AMTG, achieved moderate tumor uptake (15%IA/g at 1 h p.i., 10%IA/g at 24 h p.i.), along with manageable pancreatic retention and very low hepatic accumulation.^18^ These features, combined with the favorable emission characteristics of terbium-161, make it a promising candidate for both imaging and therapy. [^177^Lu]­Lu-RM2 showed relatively lower tumor uptake (12%IA/g at 1 h p.i. and 8%IA/g at 24 h p.i.), but with excellent clearance from the pancreas and liver.^18^ The heterodimer ^86^Y/^90^Y-[RGD-Glu-DO3A]-6-Ahx-RM2 demonstrated the lowest tumor uptake (9%IA/g at 1 h p.i. and 5%IA/g at 24 h p.i.), with minimal accumulation in nontarget tissues.^28^ The tumor uptake for RM26 analogs; [^111^In]­In-X-PEG_2_-RM2 (X = NOTA, NOTAGA, DOTA, DOTAGA) was around 3%IA/g at 4 h p.i. with a further decrease to approximately 1.5%IA/g at 24 h p.i.^29^ While this may limit its therapeutic impact, it could be advantageous for diagnostic purposes due to its favorable biodistribution profile. In summary, [^177^Lu]­Lu-LF1 emerges as one of the most promising radioligands, offering superior tumor targeting and favorable clearance kinetics. Nonetheless, agents such as [^161^Tb]­Tb-AMTG and [^177^Lu]­Lu-RM2 also present balanced biodistribution profiles that may be better suited for specific therapeutic or safety-related applications.

### In Vivo Protein Binding/Metabolic Stability Studies in Murine Plasma

At 5 and 15 min after radioligand injection, 3.2% and 2.5% of the activity, respectively, were bound to murine plasma proteins. These data very well support the biodistribution data which showed very low activity concentration in blood pool. The low degree of protein binding may be attributed to the hydrophilic nature of [^177^Lu]­Lu-LF1, as indicated by its LogD value of 2.9 ± 0.04, determined in our previous studies.^20^ HPLC analysis revealed proteolytic degradation, with three main metabolites increasing over time (Figure 3). The intact radioligand decreased from 67.6 ± 2.2% at 5 min to 38.8 ± 0.9% at 15 min p.i..

**Figure 3:**
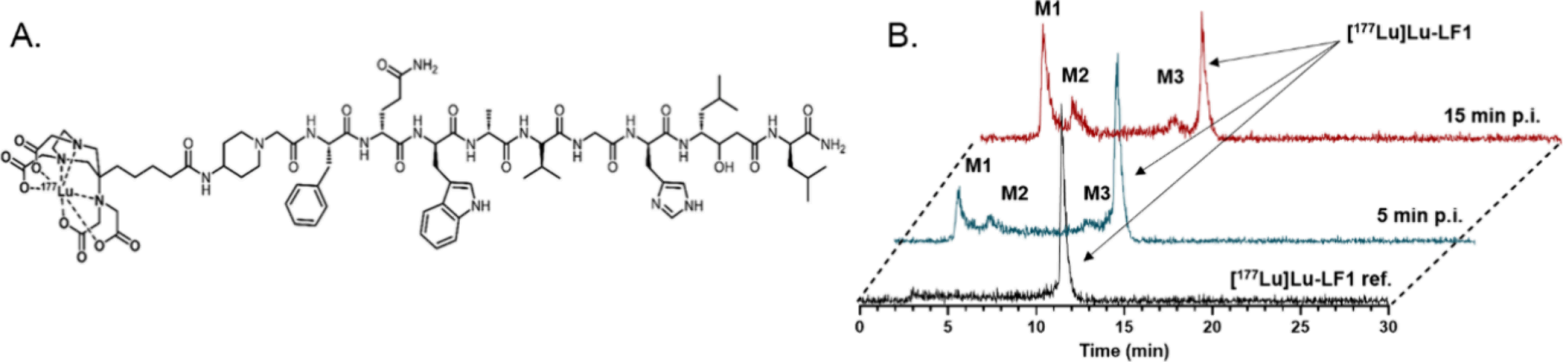
(A) Chemical structure of [^177^Lu]­Lu-LF1 and (B) representative HPLC chromatograms of blood samples collected at 5 min (blue) and 15 min (red) p.i., revealing three metabolites (M1, M2, and M3). Additionally, the reference chromatogram of [^177^Lu]­Lu-LF1 prior to its injection in PC-3 tumor-bearing mice is shown in black.

The GRPR-based antagonists are prone to proteolytic degradation, particularly by neutral endopeptidase (NEP), which can impair their in vivo targeting and theranostic efficacy.^15–17,19^ To improve their stability and performance in diagnostic imaging and radionuclide therapy, two main strategies are employed: structural modifications that retain high receptor affinity and coadministration of enzyme inhibitors to block NEP activity.^13–17,19^ However, neither approach has yet generated a GRPR-based radioantagonist with sufficient in vivo stability (Table S3).

Although the metabolites of [^177^Lu]­Lu-LF1 remain unidentified, their HPLC profiles indicate two major, early eluting species (M1 and M2), suggesting smaller, more hydrophilic fragments. Coupled with the rapid blood clearance, this supports efficient elimination and low nonspecific uptake (nontargeted organs). Similar hydrophilic metabolites were previously observed for [^68^Ga]­Ga-RM2 in both mouse and human plasma.^14,30^

### Blood and Organ Clearance Kinetics

The pharmacokinetics of [^177^Lu]­Lu-LF1 in PC-3-mice revealed critical insights into its biodistribution and clearance rates. The effective half-life of [^177^Lu]­Lu-LF1 in blood was 3.4 min in the initial distribution phase and 19.7 min in the terminal elimination phase, demonstrating its rapid systemic clearance (Figure 4A). This two-phase kinetic profile is indicative of an initial quick distribution of the radioligand into various tissues, followed by a slower elimination process likely moderated by organ-specific retention and metabolism. Such rapid blood clearance minimizes prolonged systemic exposure, which is advantageous in reducing off-target radiation exposure. The organ-specific half-life data further go deeper into the biodistribution and retention of [^177^Lu]­Lu-LF1. The pancreas exhibited an effective half-life of 1.4 h (Figure 4B), reflecting moderate retention of the radioligand, which indicates that while it is present due to the receptor-mediated process, it does not accumulate to a significant extent over prolonged periods. In contrast, the tumor exhibited a much longer effective half-life of 21.2 h (Figure 4B). This prolonged retention in tumor tissue is highly favorable for therapeutic applications. It increases the radiation dose delivered to the tumor, enhancing therapeutic efficacy while sparing normal tissues. The kidneys showed an effective half-life of 12.5 h (Figure 4B), indicating a manageable kidney exposure during the elimination process of [^177^Lu]­Lu-LF1. This pharmacokinetic and biodistribution analysis of [^177^Lu]­Lu-LF1 strongly supports its potential as a promising therapeutic agent and lays the groundwork for further clinical development aimed at improving patient outcomes.

**Figure 4:**
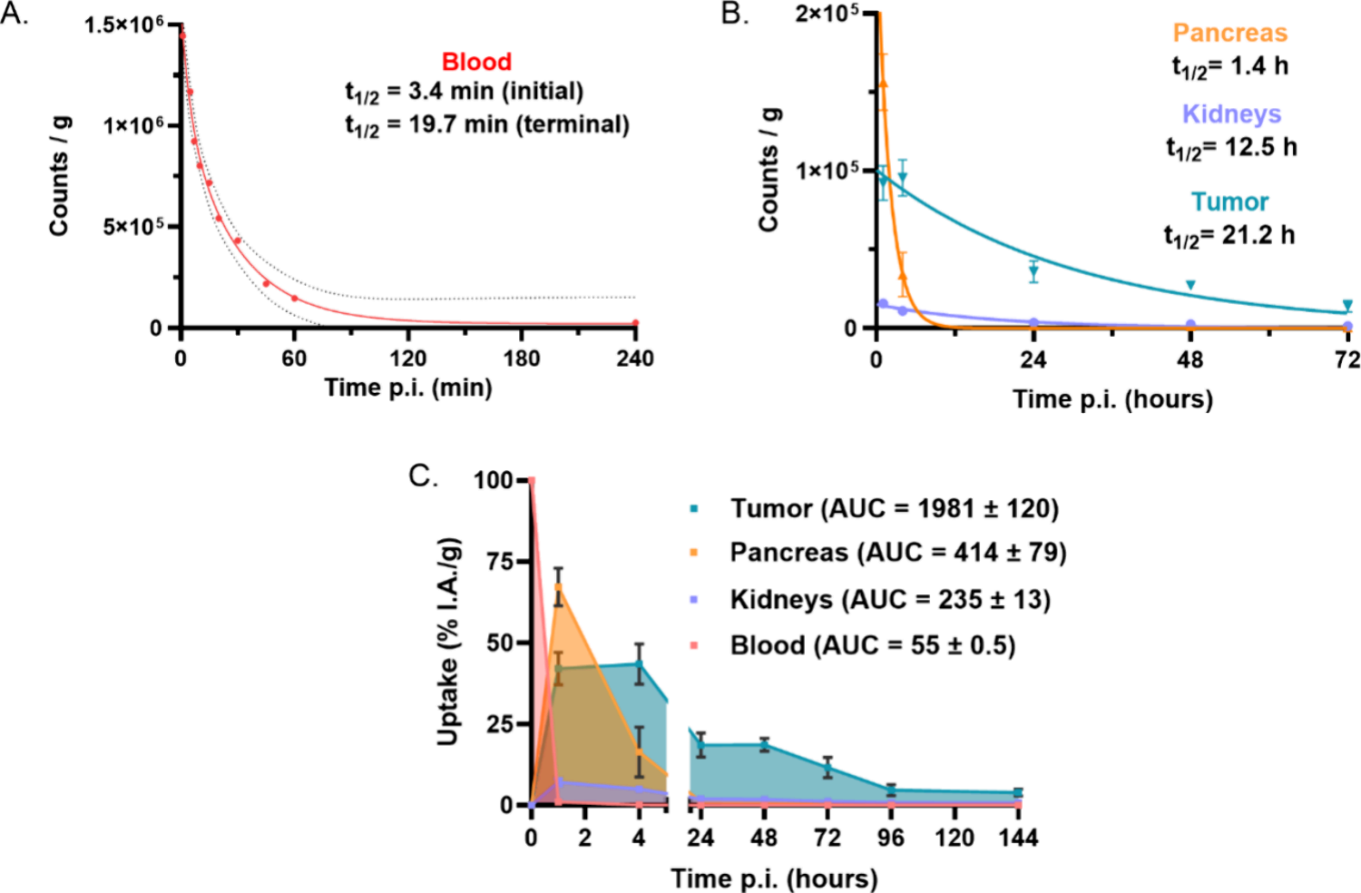
(A) Blood clearance along with the initial and terminal half-lives determined for [^177^Lu]­Lu-LF1 (*n* = 2). (B) Pharmacokinetic modeling of tumor (blue), pancreas (orange) and kidneys (violet) half-life clearance of [^177^Lu]­Lu-LF1 calculated from the biodistribution data (*n* = 3). (C) Area under the time activity curve (AUC) of [^177^Lu]­Lu-LF1 in tumor (blue), pancreas (orange), blood (red), and kidneys (violet) (*n* = 3).

The tumor clearance of [^177^Lu]­Lu-LF1, displayed in Figure 4B, seems faster than those reported for [^177^Lu]­Lu-RM2 and [^177^Lu]­Lu-NeoB in a recent side-by-side study (respectively, 40 and 50 h tumor half-life).^24^ Even so, [^177^Lu]­Lu-LF1 demonstrated higher tumor uptake values at all time points, but the different experimental conditions between the two studies (in particular regarding the animal model, doses and activities injected) must be considered when comparing these results.

### Area under the Time-Activity Curves (AUC)

The area under the time-activity curve (AUC) is a critical pharmacokinetic parameter in the development of radiotracers, targeted therapies, and diagnostic agents for cancer treatment. The AUC represents the total radioligand exposure of the tumor or a specific organ over time, and a high AUC indicates prolonged and effective accumulation of the therapeutic agent at the target site, enhancing efficacy. Figure 4C shows the AUCs from biodistribution data for tumor, blood, kidneys, and pancreas. [^177^Lu]­Lu-LF1 displays a favorable in vivo behavior, with a high and persistent tumor residency (1981 ± 120%IA/g × h), about 5 times higher than in the pancreas (414 ± 79%IA/g × h) or kidneys (235 ± 13%IA/g × h). The rather low blood AUC (55 ± 0.5%IA/g × h) matches its short half-life. These findings confirm [^177^Lu]­Lu-LF1’s strong tumor targeting, retention, and rapid clearance, supporting its promising pharmacokinetics.

### Small-Animal SPECT/CT Imaging

Representative SPECT/CT images were acquired 1, 4, 24, 48, 72, and 96 h [^177^Lu]­Lu-LF1 injection in PC-3-mice (Figure 5), showing strong agreement with biodistribution data. Fast and specific uptake in the tumor and pancreas was seen at 1 h p.i., but pancreatic activity cleared quickly, leaving mainly tumor uptake from 4 h onward and still visible at 96 h. This slower tumor washout versus rapid pancreatic clearance is consistent with previous GRPR-based tracers from our group,^25,27,31^ and may be related to the better perfusion of the pancreas compared to the tumor. Blocking experiments confirmed tumor specificity, showing reduced uptake in both tumor and pancreas on SPECT/CT.

**Figure 5:**
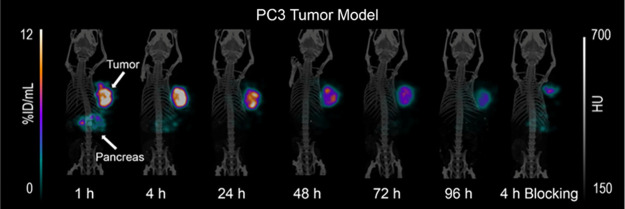
SPECT/CT images (MIP) of PC-3 tumor bearing mice (*n* = 2) upon injection of [^177^Lu]­Lu-LF1 at 1, 4, 24, 48, 72, and 96 h along with blocking studies at 4 h after injection. White arrows indicate tumor and pancreas uptake.

### In Vivo Monotherapy with [^177^Lu]­Lu-LF1

The therapeutic regimen chosen for the monotherapy study involved fractionated doses of radioactivity, a key approach in radionuclide therapy that delivers radiation in smaller, spaced-out sessions to maximize tumor killing while minimizing harm to healthy tissue. This method allows healthy cells to recover between treatments and reduces side effects.^32^

In this study, PC-3 tumors in control groups (PBS or ^nat^Lu-LF1) grew rapidly, with all mice sacrificed within 25 days (Figure 6B). Similarly, mice receiving a lower dose of [^177^Lu]­Lu-LF1 (six doses with cumulative activity 41.2 ± 0.4 MBq, 1200 pmol) showed limited tumor control and reached end points within 25 days. In contrast, the higher dose group (six doses with cumulative activity 93.4 ± 4.2 MBq, 2400 pmol) showed significant tumor suppression, with tumor volumes remaining under 400 mm^3^ for at least the first 25 days. In particular, tumor growth remained comparatively slow and controlled over an extended period, and some mice survived beyond 100 days, suggesting a long-lasting therapeutic response.

**Figure 6:**
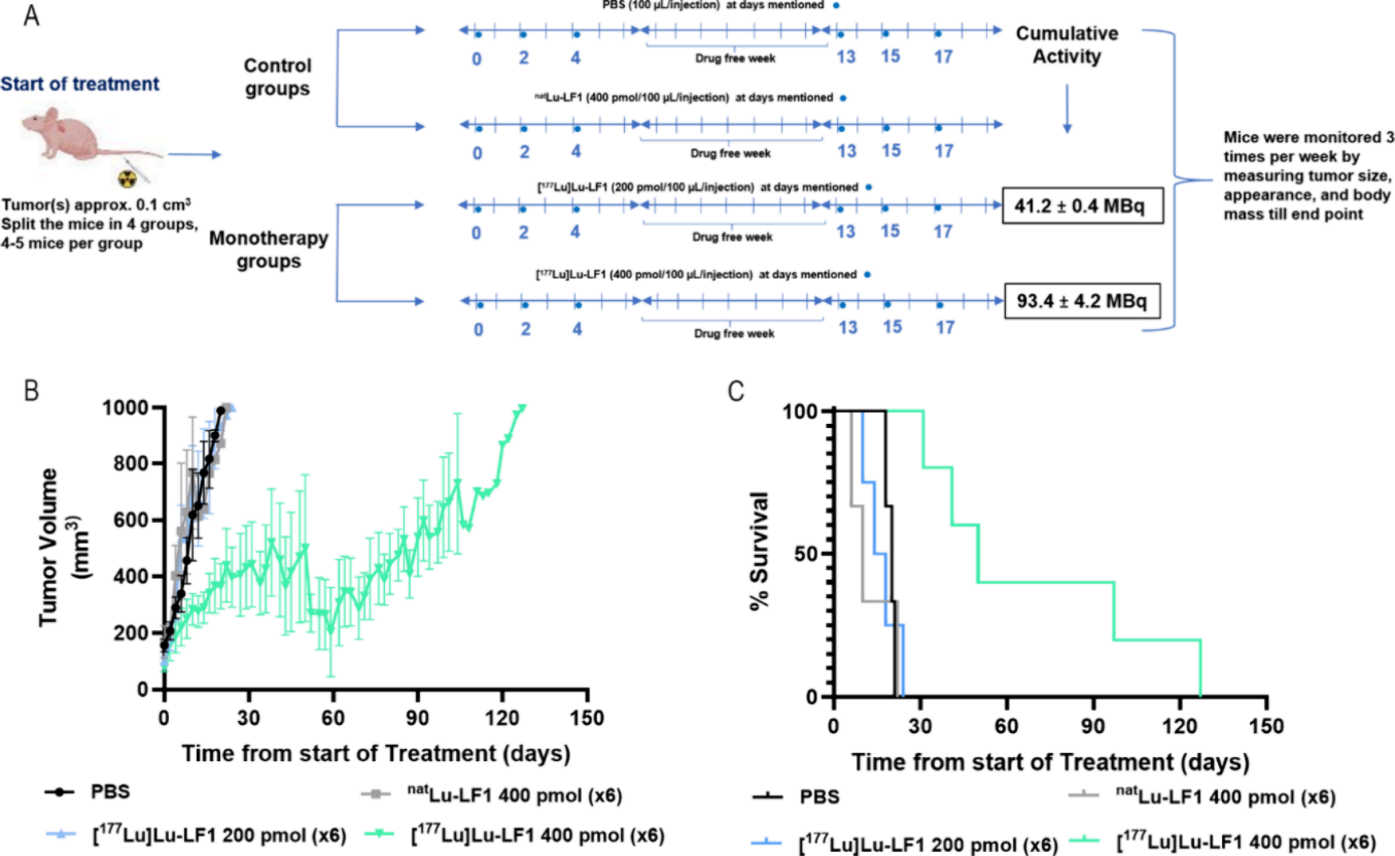
(A) Schematic representation of the monotherapy studies using [^177^Lu]­Lu-LF1 in PC-3 mice. (B) Graphical depiction of variation in the tumor volume (mm^3^) versus treatment period. (C) Survival rate in the tumor volume (mm^3^) in the different groups of monotherapy of tumor bearing mice with time.

This dosing scheme was selected based on prior biodistribution and receptor saturation studies with GRPR antagonists, which showed that this amount achieves optimal tumor targeting without saturating GRPR sites. It also falls within the well-tolerated and effective range established in previous preclinical study with [^177^Lu]­Lu-RM2.^33^

The survival analysis reflected these trends: median survival was 19, 10, and 16 days for PBS, ^nat^Lu-LF1, and low-dose groups, respectively (*p* > 0.5), while the high-dose group had a median survival of 50 days, with two mice living past 104 and 127 days. Body weight remained stable across all groups.

These results demonstrate a clear dose-dependent therapeutic effect of [^177^Lu]­Lu-LF1, with the higher dose yielding in better tumor control and extended survival.

### In Vivo Combination Therapy with [^177^Lu]­Lu-LF1 and Everolimus

The PI3K/AKT/mTOR pathway regulates cell proliferation, survival, metabolism, and angiogenesis.^34^ Its hyperactivation is common in aggressive, treatment-resistant prostate cancers, including the androgen-independent PC-3 mouse model.^35^ To target this, cotreatment with [^177^Lu]­Lu-LF1 was combined with the mTORC1 inhibitor everolimus, which blocks a key downstream node, suppressing proliferation and adaptive resistance.^34^ Previous studies showed efficacy [^177^Lu]­Lu-RM2 with rapamycin is effective in PC-3 mouse models.^33^ Everolimus, a rapamycin derivative, has better pharmacokinetics, oral bioavailability, and tissue penetration, enhancing antitumor effects in prostate and other cancers. FDA-approved for several malignancies, it has a favorable safety profile and stronger synergy with targeted therapies due to improved retention, metabolism, and immunomodulation.^36–38^

This study aimed to assess whether everolimus enhances the efficacy of [^177^Lu]­Lu-LF1 in inhibiting tumor progression. Treatment with [^177^Lu]­Lu-LF1 and/or everolimus produced varying tumor growth responses (Figure 7). PBS and ^nat^Lu-LF1 controls had rapid tumor growth, exceeding 800 mm^3^ by day 20. A single dose of [^177^Lu]­Lu-LF1 (40 ± 0.5 MBq, 400 pmol) alone did not significantly inhibit tumor growth, which progressed similarly to controls. Everolimus alone moderately delayed tumor growth but did not stop it. The greatest tumor suppression occurred with the combination therapy, where everolimus preceded [^177^Lu]­Lu-LF1 (40 ± 0.5 MBq, 400 pmol), keeping tumor volume low for over 30 days, indicating synergy.

**Figure 7:**
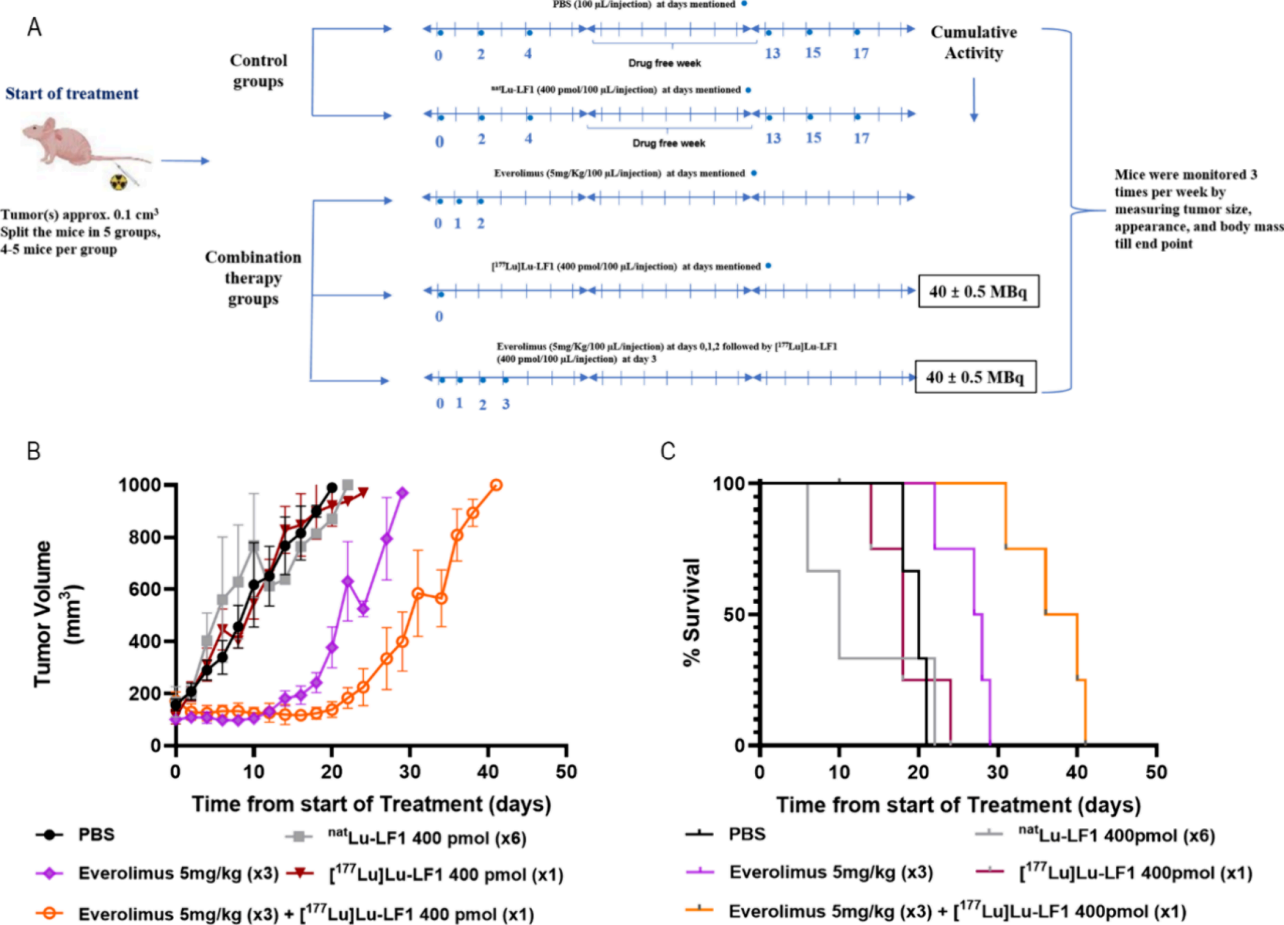
(A) Schematic representation of combination therapy study. (B) Graphical depiction of variation in the tumor volume (mm^3^) versus treatment period. (**C)** Survival rate in the different groups of combination therapy of PC-3-mice with time.

The survival analysis confirmed enhanced efficacy with combination treatment, showing a median survival of 38 days versus ∼ 14–17 days for controls (*p* < 0.01). Everolimus alone extended survival to ∼ 27 days, while a single-dose [^177^Lu]­Lu-LF1 had no significant effect (*p* > 0.5).

These findings demonstrate that combining everolimus with [^177^Lu]­Lu-LF1 therapy not only improves tumor control but also substantially prolongs survival in PC-3 tumor-bearing mice. However, in the monotherapy study, the treatment with the higher dose of [^177^Lu]­Lu-LF1 led to a longer survival compared to the combination of the lower dose of [^177^Lu]­Lu-LF1 with everolimus.

In a preclinical study, [^177^Lu]­Lu-RM2 showed promising efficacy as both fractionated monotherapy and combined with the mTOR inhibitor rapamycin. In female PC-3-bearing mice, the combination led to greater tumor growth inhibition than monotherapy.^33^ Compared to our findings, this study showed slower tumor progression and longer median survival across all groups, despite similar administered activities. These differences may be due to the gender of the animal models - Dumont et al. used females, while we used males. Additionally, the use of a different mTOR inhibitor in our study could have influenced the outcomes, a factor that warrants further investigation.

These results support the potential of combining mTOR inhibitors with radionuclide therapy to improve tumor control and highlight the need to further explore and optimize this fractionated combination approach.

### Morphological Assessment

There was no evidence of treatment-related toxicity at any of the doses administered in this study. This was evaluated through multiple parameters, including regular monitoring of animal appearance, body weight measurements taken at defined intervals, and microscopic tissue examination of organs (kidneys and pancreas) harvested at the study end point. Mice tolerated three doses of everolimus well, with no observable side effects based on physical condition or weight fluctuations. Similarly, no obvious lesions were detected in any of the collected tissues from mice treated with monotherapy and combination therapy (Table S4). These findings indicate that the dosing regimens used in both the monotherapy and combination therapy groups were safe and well tolerated.

## Conclusions

[^177^Lu]­Lu-LF1 was easily radiolabeled under mild conditions, and its AAZTA^5^ moiety offers potential for theranostic use with various radionuclide pairs. Despite the enzymatic cleavage, it showed favorable in vivo pharmacokinetics, outperforming other GRPR-targeting radioligands in clinical trials. Its high, prolonged tumor uptake and rapid clearance from blood and nontarget organs support its use in GRPR-positive tumor therapy. Fractionated regimens and combining radionuclide therapy with mTOR inhibitors present innovative strategies to enhance treatment. Overall, [^177^Lu]­Lu-LF1 is a strong candidate for targeted radionuclide therapy.

## Materials and Methods

### Radiolabeling, Quality Control, and Radiochemical Stability of [^177^Lu]­Lu-LF1

[^177^Lu]­Lu-LF1 was prepared by dissolving 5–8 μg of precursor (3–5 nmol) in 250 μL HEPES buffer (1.0 M, pH 5.4) and 20 μL of EtOH. To this solution, 30–220 MBq of [^177^Lu]­Lu^3+^ was added. The radiolabeling was complete within 10 min at room temperature. The quality control was performed by radio-TLC and HPLC. For the radiolabeling procedures performed using higher levels of radioactivity, ascorbic acid (at a final concentration of 20 μg/μL) was added postlabeling to prevent autoradiolysis. The radiochemical stability was assessed over 6 days by radio-TLC. (supplementary data)

### Binding Affinity and Binding Kinetics

For [^177^Lu]­Lu-LF1 and ligand–receptor interaction kinetics, 3 × 10^6^ PC-3 cells were seeded (supplementary data, Table S5). The association measurements were initiated with [^177^Lu]­Lu-LF1 at a final concentration of 1 nM. Once the signal reached a plateau, the concentration of [^177^Lu]­Lu-LF1 was increased to 3 nM, and the measurements continued until the second plateau was observed. At that point, the radioligand-containing medium was replaced with fresh complete medium to initiate the dissociation phase. All measurements were performed in triplicate. The resulting sensograms were analyzed using the TraceDrawer software (v 1.10, Ridgeview Instruments AB, Uppsala, Sweden) and 1–1 interaction model.

### Animal Models

Male athymic Balb/C nude mice (6 weeks old, 20–25 g; RRID:IMSR_JAX:002019) were subcutaneously implanted with PC-3 cells (3 × 10^6^ in 100 μL PBS) into the right shoulder. Once tumors reached 250–300 mm^3^ (18–20 days postimplantation), mice were used for biodistribution and SPECT/CT imaging. For all studies, mice were randomly grouped to ensure similar tumor size distribution. All procedures were approved by local authorities and conducted in accordance with institutional guidelines (license BE63/2021; 33892).

### Biodistribution Studies

Mice bearing PC-3 tumors received 10 pmol of [^177^Lu]­Lu-LF1 in 0.9% NaCl (∼0.06–0.09 MBq/0.1 mL) via tail vein injection. At 1, 4, 24, 48, 72, 96, and 144 h postinjection, animals were euthanized with an overdose of pentobarbital sodium (150 mg/kg i.p.). Selected organs were collected, weighed, and measured for radioactivity using a γ-counter. Biodistribution is expressed as % injected activity per gram (% IA/g, mean ± SD; n = 3–4). For specificity assessment, blocking studies (n = 3) were conducted by coinjecting [^177^Lu]­Lu-LF1 with 20 nmol of H-d-Phe-Gln-Trp-Ala-Val-Gly-His-Sta-Leu-NH_2_; biodistribution was assessed at 4 h p.i..

### In Vivo Protein Binding/Metabolic Stability Studies in Murine Plasma

Healthy mice (n = 2) were injected with 400 pmol of [^177^Lu]­Lu-LF1 (∼23 MBq/0.1 mL) in NaCl 0.9% and sacrificed at 5 and 15 min p.i. to determine the percentage of the radiotracer bound to the plasma proteins and the percentage of intact tracer (supplementary data).

### Blood and Organs Clearance Kinetics

Mice bearing PC-3 tumors (n = 2) received 400 pmol of [^177^Lu]­Lu-LF1 (∼23 MBq/0.1 mL in 0.9% NaCl). Blood samples were collected at multiple time points (1–240 min postinjection) and measured using a γ-counter. Blood half-lives were determined with GraphPad Prism using a Two-Phase Decay Model, while tumor, pancreas, and kidney half-lives were calculated from biodistribution data using a One-Phase Decay Model (supplementary data: Table S6).

### Area under the Time-Activity Curves (AUC)

The area under the time-activity curves (AUC) in the tumor, blood, pancreas and kidneys were calculated from the noncorrected biodistribution data using GraphPad Prism. The results of AUC are expressed as % IA/g × h and presented as mean values ± SD (n = 3) (supplementary data: Table S7).

### Small-Animal SPECT/CT Imaging

Static SPECT images (n = 2) were obtained upon injection of 400 pmol of [^177^Lu]­Lu-LF1 (∼23 MBq/100 μL) in NaCl 0.9% in the tail vein of PC-3-mice. Images were acquired at 1, 4, 24, 48, 72, and 96 h p.i.. Blocking studies (n= 2) were performed upon coinjection of 400 pmol of [^177^Lu]­Lu-LF1 and 20 nmol of H-d-Phe-Gln-Trp-Ala-Val-Gly-His-Sta-Leu-NH_2_, and the animals were imaged at 4 h p.i.. Further details on image acquisition and reconstruction are given in the supplementary data.

### In Vivo Monotherapy of PC-3 Tumor Bearing Mice Using [^177^Lu]­Lu-LF1

Tumor regression studies were conducted to evaluate the therapeutic efficacy of [^177^Lu]­Lu-LF1 in PC-3-mice using a fractionated dosing regimen (Figure 6A; Supplementary Tables S8, S10). Once average tumor volume reached 104 ± 40 mm^3^, animals were randomized into four groups (n = 4–5 per group). Two groups received [^177^Lu]­Lu-LF1 on days 0, 2, and 4, followed by a one-week treatment-free interval, and subsequently on days 13, 15, and 17. The cumulative administered activities were 41.2 ± 0.4 MBq (1200 pmol) and 93.4 ± 4.2 MBq (2400 pmol), respectively. Control groups received either ^nat^Lu-LF1 (2400 pmol) or PBS, administered according to the same schedule. Tumor volume and body weight were monitored three times weekly. Tumor volume was calculated using the formula: Width×(Length)^2^×0.5. Mice were euthanized when tumor volume exceeded 1.0 cm^3^, body weight loss surpassed 15%, or at study termination (day 150) if no end point was reached.

### In Vivo Combination Therapy with [^177^Lu]­Lu-LF1 and Everolimus

The therapeutic effect of combining [^177^Lu]­Lu-LF1 with the mTOR inhibitor everolimus was evalauted in male athymic nude mice bearing PC-3 tumors (Figure 7A; Supplementary Tables S9 and S11). Treatment began once tumors reached the same size threshold used in the monotherapy studies. Animals were divided into three groups (n = 4–5 per group) and received one of the following regimens:

1. [^177^Lu]­Lu-LF1 (40 ± 0.5 MBq, 400 pmol),
2. everolimus (5 mg/kg/day for 3 days, intraperitoneal injection), or
3. everolimus (5 mg/kg/day for 3 days, intraperitoneal injection) followed by [^177^Lu]­Lu-LF1 (40 ± 0.5 MBq, 400 pmol).

Monotherapy and combination treatments were carried out in parallel, using the same control groups (^nat^Lu-LF1, 2400 pmol, and PBS). Monitoring procedures matched those used in the monotherapy experiments.

### Morphological Assessment

Selected mouse organs (kidney, pancreas and tumor) were dissected from the sacrificed animals and immediately frozen using liquid nitrogen. Frozen tissue samples were sectioned of 7 μm thickness using a cryostat maintained at – 20 °C to – 25 °C and mounted onto microscope glass slides followed by staining with hematoxylin and eosin for microscopic examination using standard protocols (details in Supporting Information).

### Statistical Analysis

GraphPad Prism version 10.2.0 was used to performed statistical analysis using the log-rank test, with a P value of less than 0.05 considered significant.

## Supplementary Material



## Notes

Methods, results, and tables containing the individual, nonaggregated data underlying the figures, in compliance with the FAIR principles (PDF)

## Abbreviations

GRPR: gastrin-releasing peptide receptor
mTOR: mammalian target of rapamycin
SPECT/CT: single photon emission computed tomography/computed tomography
MAPK: mitogen-activated protein kinase
PI3K: phosphatidylinositol 3-kinase
PSMA: prostate-specific membrane antigen
TLC: thin-layer chromatography
HPLC: high-performance liquid chromatography
AUC: area under the curve

## References

[1] Cornelio D. B., Roesler R., Schwartsmann G. (2007). Gastrin-releasing peptide receptor
as a molecular target in experimental anticancer therapy. Ann. Oncol.

[2] Liu X., Carlisle D. L., Swick M. C., Gaither-Davis A., Grandis J. R., Siegfried J. M. (2007). Gastrin-releasing
peptide activates
Akt through the epidermal growth factor receptor pathway and abrogates
the effect of gefitinib. Exp. Cell Res..

[3] Nock B. A., Kanellopoulos P., Joosten L., Mansi R., Maina T. (2023). Peptide Radioligands
in Cancer Theranostics: Agonists and Antagonists. Pharmaceuticals (Basel).

[4] Kurth J., Krause B. J., Schwarzenböck S. M., Bergner C., Hakenberg O. W., Heuschkel M. (2020). First-in-human dosimetry of gastrin-releasing
peptide receptor antagonist [(177)­Lu]­Lu-RM2: a radiopharmaceutical
for the treatment of metastatic castration-resistant prostate cancer. Eur. J. Nucl. Med. Mol. Imaging.

[5] Mansi R., Nock B. A., Dalm S. U., Busstra M. B., van Weerden W. M., Maina T. (2021). Radiolabeled Bombesin
Analogs. Cancers (Basel).

[6] Ginj M., Zhang H., Waser B., Cescato R., Wild D., Wang X., Erchegyi J., Rivier J., Mäcke H. R., Reubi J. C. (2006). Radiolabeled somatostatin
receptor antagonists are
preferable to agonists for in vivo peptide receptor targeting of tumors. Proc. Natl. Acad. Sci. U. S. A..

[7] Liolios C., Sachpekidis C., Schäfer M., Kopka K. (2019). Bispecific radioligands
targeting prostate-specific membrane antigen and gastrin-releasing
peptide receptors on the surface of prostate cancer cells. J. Labelled Comp Radiopharm.

[8] Duan H., Song H., Davidzon G. A., Moradi F., Liang T., Loening A., Vasanawala S., Iagaru A. (2024). Prospective Comparison
of (68)­Ga-NeoB and (68)­Ga-PSMA-R2 PET/MRI in Patients with Biochemically
Recurrent Prostate Cancer. J. Nucl. Med..

[9] Minamimoto R., Sonni I., Hancock S., Vasanawala S., Loening A., Gambhir S. S., Iagaru A. (2018). Prospective Evaluation
of (68)­Ga-RM2 PET/MRI in Patients with Biochemical Recurrence of Prostate
Cancer and Negative Findings on Conventional Imaging. J. Nucl. Med..

[10] Pretze M., Reffert L., Diehl S., Schönberg S. O., Wängler C., Hohenberger P., Wängler B. (2021). GMP-compliant
production of [(68)­Ga]­Ga-NeoB for positron emission tomography imaging
of patients with gastrointestinal stromal tumor. EJNMMI Radiopharm Chem..

[11] Stoykow C., Erbes T., Maecke H. R., Bulla S., Bartholomä M., Mayer S., Drendel V., Bronsert P., Werner M., Gitsch G., Weber W. A., Stickeler E., Meyer P. T. (2016). Gastrin-releasing Peptide Receptor Imaging in Breast
Cancer Using the Receptor Antagonist (68)­Ga-RM2 And PET. Theranostics.

[12] Wong K., Sheehan-Dare G., Nguyen A., Ho B., Liu V., Lee J., Brown L., Dear R., Chan L., Sharma S., Malaroda A., Smith I., Lim E., Emmett L. (2022). (64)­Cu-SAR-Bombesin
PET-CT Imaging in the Staging of Estrogen/Progesterone Receptor Positive,
HER2 Negative Metastatic Breast Cancer Patients: Safety, Dosimetry
and Feasibility in a Phase I Trial. Pharmaceuticals
(Basel).

[13] Felber V., Holzleitner N., Joksch M., Suhrbier T., von Amsberg G., Schwarzenböck S., Kurth J., Heuschkel M., Günther T., Krause B. J. (2025). First-in-Human Serum Stability Studies
of [(177)­Lu]­Lu-AMTG: A Step Toward Improved GRPR-Targeted Radiopharmaceutical
Therapy. J. Nucl. Med..

[14] Roivainen A., Kähkönen E., Luoto P., Borkowski S., Hofmann B., Jambor I., Lehtiö K., Rantala T., Rottmann A., Sipilä H., Sparks R., Suilamo S., Tolvanen T., Valencia R., Minn H. (2013). Plasma pharmacokinetics, whole-body distribution, metabolism, and
radiation dosimetry of 68Ga bombesin antagonist BAY 86–7548
in healthy men. J. Nucl. Med..

[15] Abouzayed A., Kanellopoulos P., Gorislav A., Tolmachev V., Maina T., Nock B. A., Orlova A. (2023). Preclinical Characterization
of a Stabilized Gastrin-Releasing Peptide Receptor Antagonist for
Targeted Cancer Theranostics. Biomolecules.

[16] Chatalic K. L., Konijnenberg M., Nonnekens J., de Blois E., Hoeben S., de Ridder C., Brunel L., Fehrentz J. A., Martinez J., van Gent D. C., Nock B. A., Maina T., van Weerden W. M., de Jong M. (2016). In Vivo Stabilization of a Gastrin-Releasing Peptide
Receptor Antagonist Enhances PET Imaging and Radionuclide Therapy
of Prostate Cancer in Preclinical Studies. Theranostics.

[17] Günther T., Deiser S., Felber V., Beck R., Wester H. J. (2022). Substitution
of l-Tryptophan by α-Methyl-l-Tryptophan in (177)­Lu-RM2 Results
in (177)­Lu-AMTG, a High-Affinity Gastrin-Releasing Peptide Receptor
Ligand with Improved In Vivo Stability. J. Nucl.
Med..

[18] Holzleitner N., Cwojdzinski T., Beck R., Urtz-Urban N., Hillhouse C. C., Grundler P. V., van der Meulen N. P., Talip Z., Ramaekers S., Van de Voorde M., Ponsard B., Casini A., Günther T. (2024). Preclinical
Evaluation of Gastrin-Releasing Peptide Receptor Antagonists Labeled
with (161)Tb and (177)­Lu: A Comparative Study. J. Nucl. Med..

[19] Kanellopoulos P., Mattsson A., Abouzayed A., Obeid K., Nock B. A., Tolmachev V., Maina T., Orlova A. (2024). Preclinical evaluation
of new GRPR-antagonists with improved metabolic stability for radiotheranostic
use in oncology. EJNMMI Radiopharm Chem..

[20] Hofstetter M., Moon E. S., D’Angelo F., Geissbühler L., Alberts I., Afshar-Oromieh A., Rösch F., Rominger A., Gourni E. (2020). Effect of the versatile
bifunctional
chelator AAZTA(5) on the radiometal labelling properties and the in
vitro performance of a gastrin releasing peptide receptor antagonist. EJNMMI Radiopharm Chem..

[21] Morgan T. M., Koreckij T. D., Corey E. (2009). Targeted therapy for
advanced prostate
cancer: inhibition of the PI3K/Akt/mTOR pathway. Curr. Cancer Drug Targets.

[22] Mansi R., Wang X., Forrer F., Waser B., Cescato R., Graham K., Borkowski S., Reubi J. C., Maecke H. R. (2011). Development
of a potent DOTA-conjugated bombesin antagonist for targeting GRPr-positive
tumours. Eur. J. Nucl. Med. Mol. Imaging.

[23] Fersing C., Masurier N., Rubira L., Deshayes E., Lisowski V. (2022). AAZTA-Derived
Chelators for the Design of Innovative Radiopharmaceuticals with Theranostic
Applications. Pharmaceuticals (Basel).

[24] Damiana T. S. T., Paraïso P., de Ridder C., Stuurman D., Seimbille Y., Dalm S. U. (2023). Side-by-side comparison
of the two widely studied GRPR radiotracers, radiolabeled NeoB and
RM2, in a preclinical setting. Eur. J. Nucl.
Med. Mol. Imaging.

[25] Gourni E., Del Pozzo L., Kheirallah E., Smerling C., Waser B., Reubi J. C., Paterson B. M., Donnelly P. S., Meyer P. T., Maecke H. R. (2015). Copper-64
Labeled Macrobicyclic Sarcophagine Coupled
to a GRP Receptor Antagonist Shows Great Promise for PET Imaging of
Prostate Cancer. Mol. Pharmaceutics.

[26] Dalm S., Duan H., Iagaru A. (2024). Gastrin Releasing
Peptide Receptors-targeted
PET Diagnostics and Radionuclide Therapy for Prostate Cancer Management:
Preclinical and Clinical Developments of the Past 5 Years. PET Clin.

[27] Mansi R., Abiraj K., Wang X., Tamma M. L., Gourni E., Cescato R., Berndt S., Reubi J. C., Maecke H. R. (2015). Evaluation
of three different families of bombesin receptor radioantagonists
for targeted imaging and therapy of gastrin releasing peptide receptor
(GRP-R) positive tumors. J. Med. Chem..

[28] Bandara N., Stott Reynolds T. J., Schehr R., Bandari R. P., Diebolder P. J., Krieger S., Xu J., Miao Y., Rogers B. E., Smith C. J. (2018). Matched-pair, (86)­Y/(90)­Y-labeled, bivalent RGD/bombesin
antagonist, [RGD-Glu-[DO3A]-6-Ahx-RM2], as a potential theranostic
agent for prostate cancer. Nucl. Med. Biol..

[29] Mitran B., Varasteh Z., Selvaraju R. K., Lindeberg G., Sörensen J., Larhed M., Tolmachev V., Rosenström U., Orlova A. (2016). Selection of optimal chelator improves
the contrast of GRPR imaging using bombesin analogue RM26. Int. J. Oncol..

[30] Popp I., Del Pozzo L., Waser B., Reubi J. C., Meyer P. T., Maecke H. R., Gourni E. (2017). Approaches to improve metabolic stability
of a statine-based GRP receptor antagonist. Nucl. Med. Biol..

[31] Jamous M., Tamma M. L., Gourni E., Waser B., Reubi J. C., Maecke H. R., Mansi R. (2014). PEG spacers
of different length influence
the biological profile of bombesin-based radiolabeled antagonists. Nucl. Med. Biol..

[32] Fowler J. F. (2005). The radiobiology
of prostate cancer including new aspects of fractionated radiotherapy. Acta Oncol.

[33] Dumont R. A., Tamma M., Braun F., Borkowski S., Reubi J. C., Maecke H., Weber W. A., Mansi R. (2013). Targeted radiotherapy
of prostate cancer with a gastrin-releasing peptide receptor antagonist
is effective as monotherapy and in combination with rapamycin. J. Nucl. Med..

[34] Bjornsti M. A., Houghton P. J. (2004). The TOR pathway:
a target for cancer therapy. Nat. Rev. Cancer.

[35] Rao N. K., Shi G. P., Chapman H. A. (1995). Urokinase
receptor is a multifunctional
protein: influence of receptor occupancy on macrophage gene expression. J. Clin Invest.

[36] Spence S. L., Shaffer A. L., Staudt L. M., Amde S., Manney S., Terry C., Weisz K., Nissley P. (2006). Transformation of late
passage insulin-like growth factor-I receptor null mouse embryo fibroblasts
by SV40 T antigen. Cancer Res..

[37] Motzer R. J., Escudier B., Oudard S., Hutson T. E., Porta C., Bracarda S., Grünwald V., Thompson J. A., Figlin R. A., Hollaender N., Urbanowitz G., Berg W. J., Kay A., Lebwohl D., Ravaud A. (2008). Efficacy of everolimus in advanced
renal cell carcinoma: a double-blind, randomised, placebo-controlled
phase III trial. Lancet.

[38] Peulen H., Belderbos J., Guckenberger M., Hope A., Grills I., van Herk M., Sonke J. J. (2015). Target delineation variability and
corresponding margins of peripheral early stage NSCLC treated with
stereotactic body radiotherapy. Radiother Oncol.

