# Supplementary material for: Preclinical Evaluation of a 177Lu-Labeled Gastrin-Releasing Peptide Receptor Antagonist and Prostate Cancer Treatment with Monotherapy and in Combination with Everolimus
Source: ACS Pharmacol Transl Sci. 2025 Oct 28;9(1):59–68. doi: 10.1021/acsptsci.5c00491 (PMC12797171; doi:10.1021/acsptsci.5c00491)
Supplement: Supplementary file 1 [file pt5c00491_si_001.pdf]

SUPPLEMENTARY MATERIAL

Preclinical Evaluation of a ^{177}Lu-labeled Gastrin-Releasing Peptide Receptor Antagonist and Prostate Cancer Treatment with Monotherapy and in Combination with Everolimus

Naveen Kumar[1], Adrianna Bilinska[1,2], Elena Menéndez[1], Tilman Läppchen[1], Panagiotis Kanellopoulos[3], Anna Orlova[3], Frank Rösch[4], Axel Rominger[1], Eleni Gourni[1*]

[1]Department of Nuclear Medicine, Inselspital, Bern University Hospital, University of Bern, Bern, Switzerland 3010

[2]Graduate School of Cellular and Biomedical Sciences, University of Bern, Bern, Switzerland 3010

[3]Department of Medicinal Chemistry, Uppsala University, Uppsala, Sweden 751 83

[4]Department of Chemistry—TRIGA site, Johannes Gutenberg—University of Mainz, Germany 55128

***Corresponding author:**

Eleni Gourni

Department of Nuclear Medicine

Bern University Hospital, Switzerland

Rosenbühlgasse 25, 3010 Bern, Switzerland.

Email: eleni.gourni@insel.ch

Tel.: +41 (0)31 664 0507.

Content

S.No.	Description	Page No.
	**Methods**	
**1.**	Reagents and Instrumentation	S3
**2.**	Radiolabeling, Quality Control and Radiochemical Stability of [^{177}Lu]Lu-LF1	S3
**3.**	Binding affinity and Binding Kinetics	S4
**4.**	In Vivo Protein Binding / Metabolic Stability Studies in Murine Plasma	S4
**5.**	Blood and Organs Clearance Kinetics	S5
**6.**	Small-Animal SPECT/CT Imaging	S5
**7.**	In vivo monotherapy of PC3 tumor bearing mice using [^{177}Lu]Lu-LF1	S6
**8.**	In vivo combination therapy with [^{177}Lu]Lu-LF1 and Everolimus	S7
**9.**	Morphological assessment	S7
	**Results**	
**10.**	Quality Control and Radiochemical Stability, Figure S1, Figure S2	S8-S10
**11.**	Table S1: Biodistribution data of [^{177}Lu]Lu-LF1 in PC3 xenografts.	S11
**12.**	Table S2: Comparison of pharmacokinetics and tumor uptake between [^{177}Lu]Lu-LF1 and other GRPR antagonists	S12
**13.**	Table S3: Various strategies for improvement of metabolic stability of GRPR antagonists.	S13
**14.**	Table S4: Microscopic tissue examination for kidney, pancreas and tumor of control groups and treated group of mono therapy and combination therapy	S14
**15.**	Table S5: LigandTracer results in 1-1 model in PC-3 cells	S14
**16.**	Table S6: Raw data for organ clearance (kidney, pancreas, tumor), n=3	S15
**17.**	Table S7: Raw data for AUC (blood, kidney, pancreas, tumor), n=3	S15
**18.**	Table S8: Tumor volume (mm^3) for control and monotherapy group mice till end point, n=4-5	S16
**19.**	Table S9: Tumor volume (mm^3) for control and combination therapy group mice till end point, n=4-5	S17
**20.**	Table S10: Median survival (days) for control and monotherapy group mice	S18
**21.**	Table S11: Median survival (days) for control and combination therapy group mice	S18
**23.**	Figure S3. Schematic timeline showing treatment initiation, injection days and sacrifice points for monotherapy and combination therapy studies using [^{177}Lu]Lu-LF1 in PC-3 mice.	S18
	**References**	S19

METHODS

Reagents and Instrumentation

All reagents and solvents were obtained from Sigma-Aldrich, Merck, Fluka, VWR, AcrosOrganics and Fisher Scientific and used without further purification. [^{177}Lu]Lu^{3+} was obtained from DSD Pharma GmbH. The quality control of the radiolabeled peptide was assessed by radio Thin Layer Chromatography (radio-TLC) and by Reverse-Phase High Performance Liquid Chromatography (RP-HPLC). The radio-TLC analysis were performed with a Mini-GITA Dual TLC scanner and the TLC software Gina Star from Elysia Raytest. RP-HPLC was performed with a 1260 Infinity II LC System from Agilent equipped with an analytical 120-5 C18 Nucleosil column (250 x 4.5 mm). The elution was monitored via absorbance at 214 nm using a 1260 Infinity II Variable Wavelength Detector (VWD) G7114A and via γ-detection using a Na(Tl) well-type scintillation Gabi NOVA using the HPLC software Gina Star from Elysia Raytest.

The human prostate adenocarcinoma cell line PC3 was obtained from CLS Cell Lines Service GmbH, (Eppelheim, Germany). The Dulbecco's Modified Eagle Medium (DMEM) with GlutaMax-I Supplement, the F-12 Nutrient Mixture with GlutaMax-I Supplement, the Dulbecco's Phosphate Buffered Saline (DPBS), the Fetal Bovine Serum (FBS), the Penicillin-Streptomycin antibiotic solution and the Trypsin-EDTA were from Gibco BRL, Life Technologies (Grand Island, NY) and purchased from ThermoFisher (Switzerland). For the biodistribution and in vivo metabolic stability studies, quantitative γ-counting was performed with a Cobra II Gamma Counter from Packard Instrument (USA). For µSPECT/CT studies, a dedicated micro-PET/SPECT/CT scanner (Albira Si; Bruker Biospin, Ettlingen, Germany) was used. Mice were purchased from Charles River Laboratories (Domain des Oncins, France), pentobarbital natrium (150mg/kg) from Streuli Pharma SA (Uznach, Switzerland).

Radiolabeling, Quality Control and Radiochemical Stability of [^{177}Lu]Lu-LF1

The radiolabeling of [^{177}Lu]Lu-LF1 was performed by dissolving 5–8 µg of precursor (approximately 3–5 nmol) in 250 µL HEPES buffer (1.0 M, pH 5.4) and 20 µL of EtOH followed by the anticipated activity of [^{177}Lu]Lu^{3+} (30–220 MBq), depending on the experiment. The radiolabeling was completed

within 10 min at room temperature (r.t.). The quality control of the radiolabeling mixture was performed by radio thin-layer chromatography (TLC) and High-Performance Liquid Chromatography (HPLC) analysis, as described with details in the supplementary data. For the radiolabeling procedures involving high levels of radioactivity, ascorbic acid was added to the [^{177}Lu]Lu-LF1 radiolabeling mixture immediately after completion of the radiolabeling, to a final concentration of 20 μg/μL, in order to prevent autoradiolysis. The radiochemical stability of [^{177}Lu]Lu-LF1 was evaluated for a period of 6 days by radio-TLC analysis.

Binding affinity and Binding Kinetics

For the in vitro ligand-receptor interaction kinetic evaluation of [^{177}Lu]Lu-LF1, 3 × 10^6 PC-3 cells were seeded the day prior of the experiment. Cells were suspended in 4-5 mL of complete medium and seeded in one part of a 10 cm plastic petri dish, while keeping the dish in an angle. The day of the experiment, supernatant was aspirated and the dish was washed gently with 4 mL of media and the liquid was removed by aspiration. Next, 8-10 mL of complete media were introduced and the dish with the cells was left to incubate at 37°C for 0.5-1 h. Then, the medium was removed and the cells were placed in the holder of a LigandTracer White or Grey (Ridgeview Instruments AB, Uppsala, Sweden). New media (3 mL) were introduced and background measurements were conducted for approximately 20-30 min, then the measurements of the kinetics were performed as stated in the "Materials and Methods" section of the manuscript.

In Vivo Protein Binding / Metabolic Stability Studies in Murine Plasma

Healthy mice (n = 2) were injected with 400 pmol of [^{177}Lu]Lu-LF1 (~23 MBq / 0.1 mL) in NaCl 0.9% and sacrificed at 5 and 15 min post-injection (p.i.). Blood was immediately transferred to pre-chilled heparinized tubes and centrifuged (5 min, 1700 g, 4 °C) for plasma isolation. The isolated plasma was then transferred into a 2 mL Eppendorf and a double volume of a 1:1 (v/v) ACN:MeOH solution was added to induce the precipitation of the plasma proteins. The proteins were separated by centrifugation (10 min, 9660 g, 4 °C). After careful separation of the two phases (precipitated proteins and supernatant), the respective activities were measured in a γ-counter to determine the percentage of the radiotracer

bound to the plasma proteins.

To evaluate the in vivo metabolic stability of [^{177}Lu]Lu-LF1 in blood circulation and determine if and to which extent the remaining circulating activity in blood is subjected to enzymatic degradation, samples from the supernatant were analyzed by radio-HPLC (from both tested time points 5 and 15 min p.i.). The same conditions which have been described for the quality control of [^{177}Lu]Lu-LF1 were used also for the analysis of the radio-metabolites.

Blood and Organs Clearance Kinetics

Mice bearing PC3 tumors (n= 2) were injected with 400 pmol of [^{177}Lu]Lu-LF1 (~23 MBq/0.1 mL) in NaCl 0.9%. Blood samples were withdrawn from the facial vein at 1, 3, 5, 7, 10, 15, 20, 30, 45, 60 and 240 min p.i.. Blood was transferred in pre-weighted capillary tubes and the radioactivity was counted in a γ-counter. The initial and terminal half-lives of [^{177}Lu]Lu-LF1 in blood were determined using GraphPad Prism 10.2.0 software, employing the non-linear regression Two-Phase Decay Model. The half-lives of [^{177}Lu]Lu-LF1 in tumor, pancreas and kidney were calculated accordingly, using the biodistribution data and plotting them against time, employing the non-linear regression One-Phase Decay Model.

Small-Animal SPECT/CT Imaging

Static SPECT images were obtained upon injection of 400 pmol of [^{177}Lu]Lu-LF1 (~23 MBq/100 μL) in NaCl 0.9% in the tail vein of PC3-mice. Images were acquired at 1, 4, 24, 48, 72 and 96 h p.i. and imaging was performed in spontaneously breathing animals under isoflurane anesthesia (2 % isoflurane, 1.5 mL/min oxygen). Blocking studies (n = 2) were performed upon co-injection of 400 pmol of [^{177}Lu]Lu-LF1 and 20 nmol of the blocking agent H-D-Phe-Gln-Trp-Ala-Val-Gly-His-Sta-Leu-NH_2, and the animals were imaged at 4 h p.i. under the same conditions as described above.

SPECT images were acquired using a 208 keV ± 20 % energy window and a Cerrobase (Bi-Pb alloy) 30 mm thick collimator, with a pinhole 22 mm thick tungsten lens, 60 projections and 60 – 1440 s per projection. SPECT data were reconstructed using the Ordered Subset Expectation Maximization (OSEM) algorithm with 2 iterations of 5 subsets and a voxel size of 0.5 mm. The reconstructed data

were corrected for lutetium-177 decay, normalized and filtered, using a Gaussian 3D algorithm with a 1.5 mm isotropic kernel, and generated using PMOD software The color scale of the SPECT images was set from 0 to 12 % I.A./mL, allowing for qualitative comparison among the images. The CT was carried out using step-and-shoot mode, employing 45 kVp and 400 μA as settings. CT data were reconstructed using FBP algorithm and a voxel size of 0.125 mm. The color scale of the CT is ranging from 150 to 700 HUs.

In vivo monotherapy of PC3 tumor bearing mice using [^{177}Lu]Lu-LF1

Tumor regression studies were carried out in order to evaluate the therapeutic efficacy of [^{177}Lu]Lu-LF1. A fractionated dosing scheme was applied in male athymic nude mice subcutaneously implanted with PC3 tumors (Figure 6A).

When the average tumor size at the start of treatment reached approximately 104 ± 40 mm3, the mice were divided in 5 groups with 5 mice each group. Two groups received 3 injections of [177Lu]Lu-LF1 per week on days 0, 2, and 4 of therapy. After a one-week drug-free interval, the same procedure was repeated on days 13, 15, and 17. The total administered activity for these groups was 41.2 ± 0.4 MBq (in total 1200 pmol) and 93.4 ± 4.2 MBq (in total 2400 pmol), delivered in doses of 6–7 MBq and 14–15 MBq (corresponding to 200 and 400 pmol per dose, respectively). Two control groups were included in the study: one received ^{nat}Lu-LF1(in total 2400 pmol) in 100 μL of NaCl 0.9%, and the other received PBS (100 μL). Both control groups were administered their respective treatments on the same days as the [^{177}Lu]Lu-LF1-treated groups. The mice were monitored 3 times per week by measuring tumor size and their body weight. As ending point was set when tumors reach a volume of 1.0 cm3 or 150 days if the therapy is successful and the above tumor size has not been reached. Tumor size was measured with calipers in 2 dimensions, and tumor volume was calculated assuming an elliptical shape. Tumor volume at each time point was calculated as: Width × (Length)2 × 0.5. Body weight of the animals was measured 3 times per week to observe the acute toxicity associated with the treated groups compared to the control animals. Mice were euthanized if they experienced a weight loss exceeding 15% of their initial body weight or if tumor volume surpassed 1.0 cm^3 prior to the study endpoint.

In vivo combination therapy with [^{177}Lu]Lu-LF1 and Everolimus

The in vivo evaluation of the therapeutic efficacy of combination therapy using [^{177}Lu]Lu-LF1 and an mTOR inhibitor, everolimus, was conducted on male athymic nude mice bearing PC3 xenografted tumors (Figure 7A). The procedure is detailed in the supplementary material.

Consistent with the monotherapy studies, identical criteria for tumor size were applied to determine the initiation of therapy in the combination treatment studies. Three groups of 5 mice per group were subjected to the following treatment regimens: (a) administration of [^{177}Lu]Lu-LF1 (40 ± 0.5 MBq, 400 pmol). (b) Treatment with the mTOR inhibitor everolimus at a dose of 5 mg/kg/day for a duration of 72 h. (c) A combination regimen where everolimus was administered at 5 mg/kg/day for three consecutive days, followed by [^{177}Lu]Lu-LF1 (40 ± 0.5 MBq, 400 pmol). Both monotherapy and combination therapy studies conducted in parallel; consequently, the control groups receiving ^{nat}Lu-LF1 (in total 2400 pmol) and PBS served as shared controls for both experiments. The animals were monitored throughout the combination therapy study using the same methodology previously described for the monotherapy studies.

Morphological assessment

Frozen tissue samples (kidney, pancreas and tumor) were sectioned at 7 µm thickness using a cryostat maintained at −20°C to −25°C and mounted onto microscope glass slides. Sections were air-dried at room temperature for 15 minutes and subsequently fixed in 4% paraformaldehyde for 10 minutes. After rinsing in phosphate-buffered saline, slides were stained with hematoxylin for 5 minutes, rinsed in running tap water and differentiated in 1% acid alcohol followed by bluing in ammonia water. Sections were then counterstained with eosin for 1 minute and briefly washed in 70% ethanol to remove excess stain. Slides were dehydrated through graded ethanol (70%, 95%, 100%) and mounted with a permanent mounting medium. Stained sections were examined using a light microscope.

RESULTS

Quality Control and Radiochemical Stability

Radio-TLC analysis was performed with aluminum coated silica gel 60 F254 plates from Merck as stationary phase and citrate buffer 0.1 M at pH 5 as a mobile phase. In this chromatographic system, the radiolabeled peptide remains immobilized at the starting point, while free lutetium-177 moves with the mobile phase.

RP-HPLC analysis was performed using a linear gradient of eluent A (0.1 % (v/v) TFA in water) and eluent B (0.1 % (v/v) TFA in acetonitrile) applied according to the following method: 0-3 min (15 % B), 3-21 min (15-90 % B), 21-25 min (90 % B), 25-25.1 min (90-15 % B) and 25.1-30 (15% B).

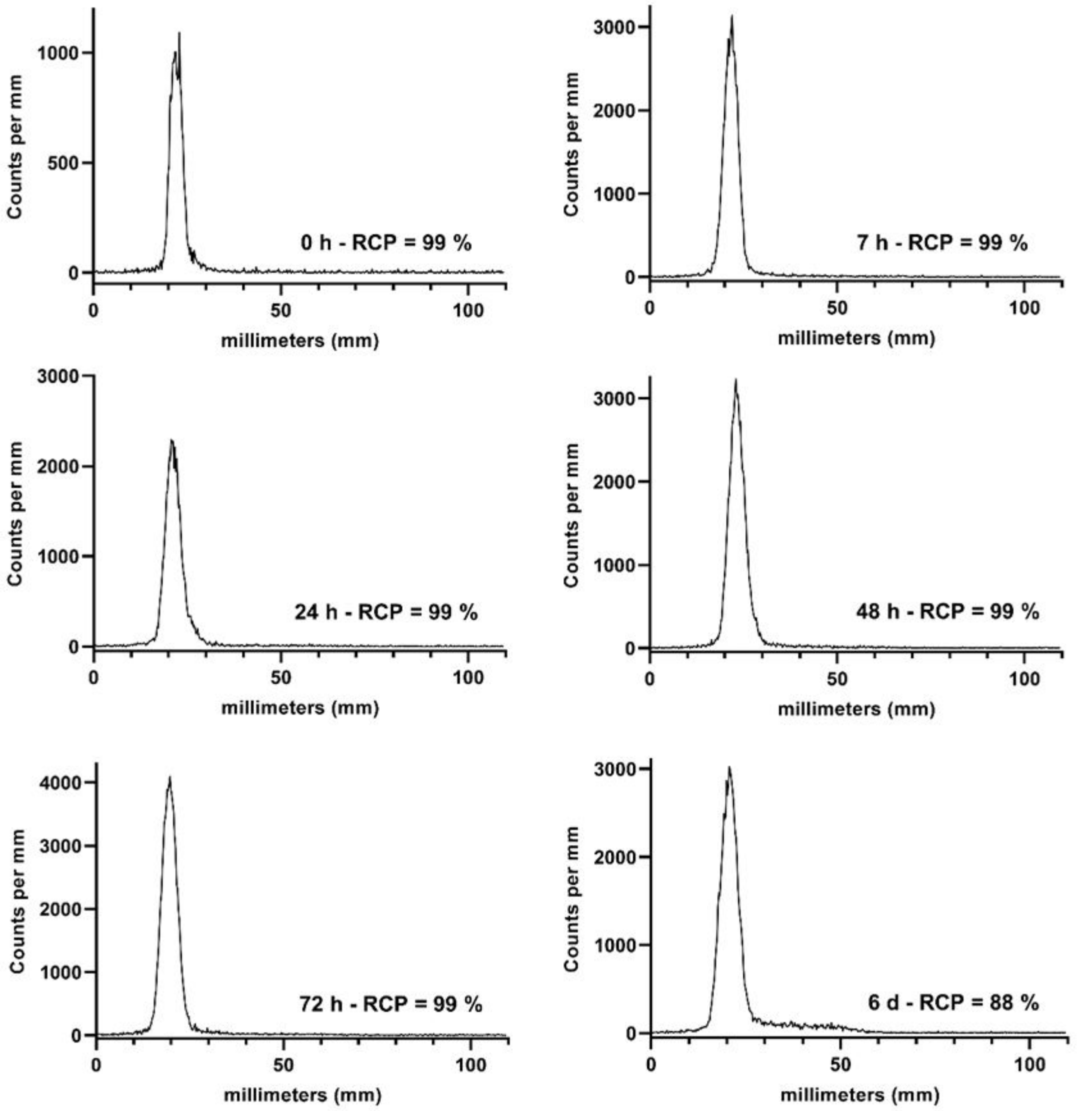

Figure S1. Radio-TLC profiles of [^{177}Lu]Lu-LF1 ($R_f = 0$) indicating the radiochemical purity (RCP) at several time points after radiolabeling (0, 7, 24, 48, 72 h and 6 d) expressed as percentage of intact tracer detected over total radioactivity.

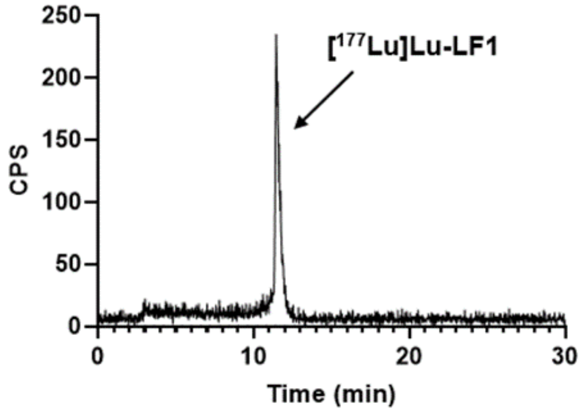

Figure S2. Radio-HPLC profile of [^{177}Lu]Lu-LF1 (5 min post labeling) performed using a linear gradients of eluent A (0.1 % (v/v) TFA in water) and eluent B (0.1 % (v/v) TFA in acetonitrile) applied according to the following method: 0-3 min (15 % B), 3-21 min (15-90 % B), 21-25 min (90 % B), 25-25.1 min (90-15 % B) and 25.1-30 (15% B).

Biodistribution Studies

Table S1: Biodistribution data of [^{177}Lu]Lu-LF1 in PC3 xenografts.

Organ	1 h	4 h	24 h	48 h	72 h	96 h	144 h	4 h blocked
Blood	1.0±0.2	0.2±0.0	0.0±0.0	0.0±0.0	0.0±0.0	0.0±0.0	0.0±0.0	0.0±0.0
Heart	0.3±0.1	0.1±0.1	0.0±0.0	0.0±0.0	0.0±0.0	0.0±0.0	0.0±0.1	0.1±0.0
Liver	0.7±0.1	0.6±0.1	0.2±0.0	0.3±0.1	0.2±0.0	0.2±0.0	0.3±0.0	0.6±0.0
Spleen	0.9±0.4	0.3±0.0	0.2±0.1	0.2±0.1	0.1±0.1	0.2±0.1	0.3±0.0	0.2±0.0
Lung	0.8±0.2	0.3±0.1	0.1±0.1	0.1±0.1	0.0±0.1	0.0±0.1	0.2±0.0	0.2±0.0
Kidneys	7.1±1.1	4.9±0.6	1.7±0.2	1.4±0.2	0.9±0.3	0.9±0.1	0.8±0.3	10±1.0
Stomach	5.4±0.7	2.8±0.7	0.1±0.0	0.1±0.0	0.0±0.1	0.0±0.0	0.0±0.1	0.3±0.1
Intestines	2.9±0.5	1.0±0.2	0.1±0.0	0.1±0.0	0.0±0.0	0.0±0.0	0.0±0.0	0.2±0.0
Adrenal	5.0±0.3	1.9±1.0	1.2±0.9	0.0±0.4	1.9±2.1	2.3±0.7	4.1±1.5	0.0±0.1
Pancreas	71±8.1	15±6.4	0.9±0.20	0.8±0.0	0.3±0.1	0.7±0.1	0.4±0.0	0.3±0.1
Muscle	0.2±0.0	0.1±0.0	0.1±0.0	0.1±0.0	0.1±0.0	0.0±0.0	0.0±0.1	0.0±0.0
Bone	0.6±0.28	0.5±0.4	0.2±0.1	0.4±0.4	0.0±0.5	0.2±0.0	0.4±0.3	0.5±0.2
Tumor	42±5.0	43±5.0	18±3.3	15±1.3	8.5±2.3	5.7±0.1	3.9±1.1	13±0.5

Data are expressed in percentage of injected activity per gram of tissue (% IA/g) and are presented as the mean values ± SD (n=3–4).

Table S2: Comparison of pharmacokinetics and tumor uptake between [^{177}Lu]Lu-LF1 and other GRPR antagonists

Tracer	Tumor uptake at 1 and 24h (%IA/g)	Pancreas uptake at 1 and 24h (%IA/g)	Kidney uptake at 1 and 24h (%IA/g)	Liver uptake at 1 and 24h (%IA/g)	Mice strain, sex	Reference
[^{177}Lu]Lu-LF1	42 and 18 ($t_{1/2}$=21.8h)	70 and 0.9 ($t_{1/2}$=1.4h)	7 and 1.7	0.7 and 0.2	Male Athymic Balb/C nude	Present Study
[^{177}Lu]Lu-NeoB	9 and 8 ($t_{1/2}$=50h)	30 and 4 ($t_{1/2}$=7.2h)	3.4 and 0.1	2.7 and 0.1	Male NMRI-Foxn1 nu/nu	1
[^{177}Lu]Lu-AMTG	14 and 11	24 and 0.5	4 and 1.2	0.7 and 0.1	Female CB17-SCID	2
[^{161}Tb]Tb-AMTG	15 and 12	42 and 1.5	3.5 and 1.5	7 and 1.5	Female CB17-SCID	2
[^{161}Tb]Tb-RM2	12 and 9	28 and 1	2.8 and 1	5.4 and 2	Female CB17-SCID	2
[^{177}Lu]Lu-RM2	12 and 8 ($t_{1/2}$=40h)	15 and 0.5 ($t_{1/2}$=1.1h)	3.7 and 1.8	1 and 0.5	Female CB17-SCID	2
^{86}Y/^{90}Y-[RGD-Glu-[DO3A]-6-Ahx-RM2]	9 and 5	7 and 0.3	2.3 and 0.9	0.4 and 0.3	Male, ICR-SCID	3
[^{111}In]In-NOTA-PEG_2-RM26*	2.5 and 1.7*	2.2 and 0.1*	3.6 and 1.3*	2.4 and 1.5*	Female BALB/c nu/nu	4
[^{111}In]In-NODAGA-PEG_2-RM26*	3.6 and 2.7*	16 and 0.2*	6.5 and 2.6*	1.2 and 0.4*	Female BALB/c nu/nu	4
[^{111}In]In-DOTA-PEG_2-RM26 *	3.4 and 1.3*	0.2 and 0.01*	2.4 and 0.6*	1 and 0.3*	Female BALB/c nu/nu	4
[^{111}In]In-DOTAGA-PEG_2-RM26*	2.3 and 1.4*	0.1 and 0.02*	2.9 and 1.7*	0.6 and 0.4*	Female BALB/c nu/nu	4

*Tumor, pancreas, kidney and liver uptake for RM26 at 4h and 24h

In Vivo Protein Binding / Metabolic Stability Studies in Murine Plasma

Table S3: Various strategies for improvement of metabolic stability of GRPR antagonists.

Peptide / Radioligand	Stabilization Strategy	In Vivo Stability	Tumor Uptake (%ID/g)	Reference
[^{68}Ga]Ga-NMe-RM2 and	Direct (Structure modification), Indirect (Co-injection with a NEP inhibitor)	Intact radiotracer (~55%) and three metabolites	13-14% IA/g tumor uptake at 1 h post-injection	5
[^{111}In]In-AU-RM26-M1	Direct (Structure modification)	Increased from 69% to 88% intact at 5 min post-injection	Increased from 5.7 to 7.0 at 1 h post-injection	6
[^{68}Ga]/[^{177}Lu]-JMV4168	Indirect (Co-injection with a NEP inhibitor)	Enhanced metabolic stability in murine blood	Increased from 9 to 19 % IA/g (^{68}Ga-JMV4168) and from 9 to 17 % IA/g (^{177}Lu-JMV4168) upon co-injection of NEP at 1 h post-injection	7
[^{111}In]In-AU-SAR-M1	Direct and indirect(Structure modification followed by inhibitor injection)	High stability; further increased with NEP inhibition	11% IA/g and 6.3 % IA/g tumor uptake at 4 h and 24 h post-injection	8
[^{177}Lu]Lu-AMTG and [^{177}Lu]Lu-AMTG2	Direct (Structure modification)	Enhanced metabolic stability in murine blood AMTG (92.9%), AMTG2 (77.6%),	11.5% IA/g and 8.0 % IA/g tumor uptake at 24 h post-injection for[^{177}Lu]Lu-AMTG and [^{177}Lu]Lu-AMTG2 respectively	9, 10

Morphological assessment

Table S4: Microscopic tissue examination for kidney, pancreas and tumor of control groups and treated group of mono therapy and combination therapy

	**Kidney**	**Pancreas**	**Tumor**
**PBS**	No obvious lesions	No obvious lesions	Multifocal to coalescing large areas of necrosis, granulocytic infiltrates, and hemorrhage
**^{nat}Lu-LF1 400 pmol (x6)**	No obvious lesions	No obvious lesions	Multifocal small areas of necrosis, granulocytic infiltrates, and hemorrhage
**[^{177}Lu]Lu-LF1 200 pmol (x6)**	No obvious lesions	No obvious lesions	Multifocal small areas of necrosis and granulocytic infiltrates.
**[^{177}Lu]Lu-LF1 LF1 400 pmol (x6)**	No obvious lesions	No obvious lesions	Multifocal small areas of necrosis and granulocytic infiltrates
**Everolimus 5mg/kg (x3)**	Vacuolar degeneration of some tubular epithelial cells. No other obvious lesions	No obvious lesions.	Multifocal small areas of necrosis and granulocytic infiltrates
**[^{177}Lu]Lu-LF1 400 pmol (x1)**	No obvious lesions	No obvious lesions	Multifocal to coalescing large areas of necrosis, granulocytic infiltrates, and hemorrhage. Multifocal large lymphocytic aggregates
**Everolimus 5mg/kg (x3) [^{177}Lu]Lu-LF1 400 pmol (x1)**	Vacuolar degeneration of some tubular epithelial cells. No other obvious lesions	No obvious lesions	Multifocal small areas of necrosis and granulocytic infiltrates

Table S5: LigandTracer results in 1-1 model in PC-3 cells

**Binding kinetics**					
**Repetition**	**1**	**2**	**3**	**Average**	**SD**
**k_{on} ($M^{-1} \times s^{-1}$)**	2.40×10^{5}	2.67×10^{5}	2.36×10^{5}	2.48×10^{5}	16862.18649
**k_{off} ($M^{-1} \times s^{-1}$)**	3.12×10^{-5}	3.19×10^{-5}	3.29×10^{-5}	3.20×10^{-5}	8.544×10^{-7}
**K_D (M)**	1.27×10^{-10}	1.19×10^{-10}	1.40×10^{-10}	1.23×10^{-10}	1.05987×10^{-11}

Table S6: Raw data for organ clearance (kidney, pancreas, tumor), n=3

Time p.i.	Kidney (Counts/g)			Pancreas (Counts/g)			Tumor (Counts/g)		
	**Mice 1**	**Mice 2**	**Mice 3**	**Mice 1**	**Mice 2**	**Mice 3**	**Mice 1**	**Mice 2**	**Mice 3**
**1 h**	15327.8	18404.1	13536.9	155423.7	174704.7	138960.7	87595.3	104921.4	84400.8
**4 h**	9144	10486.6	12269.3	19307.9	29402.8	52930.1	78819.5	103604.5	101336.9
**24 h**	3970	3357	2814.5	1504.7	1494.5	1698.6	41806.6	29705.6	30255.6
**48 h**	2673.3	2579.9	2086.4	1298.3	1369.4	1441.4	25283.2	30735.6	26227.4
**72 h**	1762.2	884.1	1727.4	681	366.7	660.5	14093.1	10206.7	17774.6

Table S7: Raw data for AUC (blood, kidney, pancreas, tumor), n=3

Time p.i.	Blood (%IA/g)			Kidney (%IA/g)			Pancreas (%IA/g)			Tumor (%IA/g)		
	Mice 1	Mice 2	Mice 3	Mice 1	Mice 2	Mice 3	Mice 1	Mice 2	Mice 3	Mice 1	Mice 2	Mice 3
**0 h**	100	100	100	0	0	0	0	0	0	0	0	0
**1 h**	0.98	1.22	0.92	7.02	8.37	6.14	71.26	63.09	68.09	40.16	47.71	38.32
**4 h**	0.12	0.15	0.18	4.21	4.81	5.64	8.90	24.34	15.97	36.33	47.56	46.60
**24 h**	0.01	0.01	0.01	2.17	1.83	1.53	0.82	0.92	1.26	22.94	16.26	16.51
**48 h**	0.00	0.01	0.01	1.81	1.75	1.41	0.88	0.97	0.99	17.16	20.85	17.79
**72 h**	0.00	0.00	0.00	1.46	0.73	1.43	0.56	0.54	0.55	11.68	8.46	14.73
**96 h**	0.00	0.00	0.01	0.92	0.93	0.73	0.70	0.51	0.62	5.66	5.74	2.64
**144 h**	0.00	0.00	0.00	0.98	0.62	0.75	0.42	0.45	0.32	4.68	3.11	3.97

Table S8: Tumor volume (mm^3) for control and monotherapy group mice till end point, n=4-5

**Time p.i. (Days)**	**PBS**		**^{nat}Lu-LF1 400 pmol (x6)**		**[^{177}Lu]Lu-LF1 200 pmol (x6)**		**[^{177}Lu]Lu-LF1 LF1 400 pmol (x6)**	
	**Average**	**S.D.**	**Average**	**S.D.**	**Average**	**S.D.**	**Average**	**S.D.**
0	156.09	22.34	168.13	57.93	101.54	15.15	78.39	15.09
2	207.85	31.80	204.26	25.57	185.31	47.17	158.28	56.58
4	289.13	38.59	402.48	106.21	284.36	42.23	187.50	58.17
6	339.08	64.67	559.89	240.50	534.00	118.30	220.88	65.76
8	456.12	81.61	627.72	219.32	533.02	73.66	250.52	69.64
10	616.96	162.24	767.90	200.00	727.63	134.32	283.49	55.70
12	650.15	114.96	611.90		674.65	168.16	276.29	55.80
14	766.76	110.47	638.14		765.40	160.45	289.53	55.587
16	815.14	103.25	763.90		786.66	164.22	340.35	69.59
18	899.07	22.73	813.84		890.60	110.32	365.86	97.16
20	990.50	10.02	870.25		932.65		365.24	80.22
22			997.50		973.35		438.93	129.79
24					999.20		396.67	106.10
27							405.90	146.27
29							432.64	147.72
31							442.41	150.55
34							376.50	111.78
36							426.29	155.27
38							519.64	189.38
41							458.95	200.78
43							366.50	173.61
45							416.17	211.10
48							469.47	232.85
50							500.35	259.83
Till 100 days							644.42	177.61
End point 127 days							997.50	

Table S9: Tumor volume (mm^3) for control and combination therapy group mice till end point, n=4-5

Time p.i. (Days)	PBS		^{nat}Lu-LF1 400 pmol (x6)		Everolimus 5mg/kg (x3)		[^{177}Lu]Lu-LF1 400 pmol (x1)		Everolimus 5mg/kg (x3) [^{177}Lu]Lu-LF1 400 pmol (x1)	
	Average	S.D.	Average	S.D.	Average	S.D.	Average	S.D.	Average	S.D.
0	156.09	22.34	168.13	57.93	100.06	15.54	111.07	28.07	171.15	34.97
2	207.85	31.80	204.26	25.57	108.59	6.98	207.97	37.74	129.28	31.21
4	289.13	38.59	402.48	106.21	107.95	21.62	311.14	64.16	125.43	29.11
6	339.08	64.67	559.89	240.50	97.61	16.23	445.83	78.61	132.49	22.10
8	456.12	81.61	627.72	219.32	97.18	5.02	402.95	28.50	132.43	30.44
10	616.96	162.24	767.90	200.00	104.48	6.16	551.49	65.87	124.44	21.61
12	650.15	114.96	611.90		131.10	16.63	657.27	58.01	125.79	36.29
14	766.76	110.47	638.14		181.07	29.10	827.94	89.97	119.52	38.04
16	815.14	103.25	763.90		193.30	34.93	846.56	120.09	116.77	17.02
18	899.07	22.73	813.84		241.75	38.15	896.36	103.75	124.08	22.43
20	990.50	10.02	870.25		376.61	78.93	920.93	79.29	138.52	29.77233
22			988.90		630.33	151.97	937.69		182.89	39.55
24					524.51	30.28	970.50		223.97	71.20
27					794.32	157.87			333.82	118.79
29					970.40	14.18			398.98	112.99
31									584.60	165.27
34									565.33	109.22
36									807.78	99.90
38									893.26	52.4751
41									998.28	

Table S10: Median survival (days) for control and monotherapy group mice, n=4-5

Groups	PBS	^{nat}Lu-LF1 400pmol (X6)	[^{177}Lu]Lu-LF1 200pmol (X6)	[^{177}Lu]Lu-LF1 400pmol (X6)
Median survival (days)	20	10	16	50

Table S11: Median survival (days) for control and combination therapy group mice, n=4-5

Groups	PBS	^{nat}Sc-LF1 400 pmol	[^{177}Lu]Lu-LF1 400pmol (X1)	Everolimus 5mg/Kg (X3)	Everolimus 5mg/Kg (X3) + [^{177}Lu]Lu-LF1 400pmol (X1)
Median survival (days)	20	10	18	27.5	38

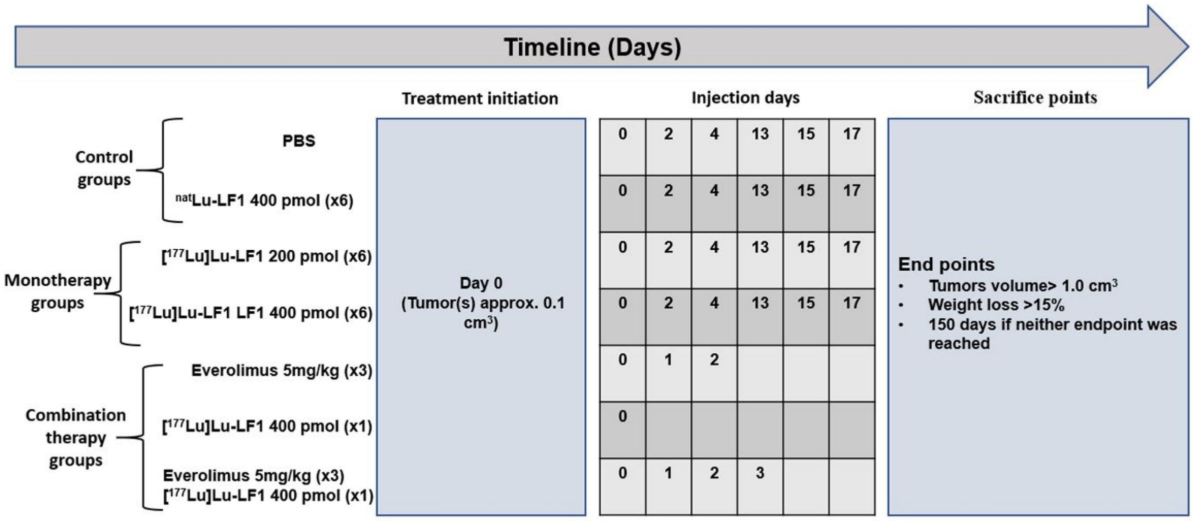

Figure S3. Schematic timeline showing treatment initiation, injection days and sacrifice points for monotherapy and combination therapy studies using [^{177}Lu]Lu-LF1 in PC-3 mice.

References

(1) Damiana, T. S. T.; Paraïso, P.; de Ridder, C.; Stuurman, D.; Seimbille, Y.; Dalm, S. U. Side-by-side comparison of the two widely studied GRPR radiotracers, radiolabeled NeoB and RM2, in a preclinical setting. *Eur J Nucl Med Mol Imaging* **2023**, *50* (13), 3851-3861. DOI: 10.1007/s00259-023-06364-4.
(2) Holzleitner, N.; Cwojdzinski, T.; Beck, R.; Urtz-Urban, N.; Hillhouse, C. C.; Grundler, P. V.; van der Meulen, N. P.; Talip, Z.; Ramaekers, S.; Van de Voorde, M.; et al. Preclinical Evaluation of Gastrin-Releasing Peptide Receptor Antagonists Labeled with (161)Tb and (177)Lu: A Comparative Study. *J Nucl Med* **2024**, *65* (3), 481-484. DOI: 10.2967/jnumed.123.266233.
(3) Bandara, N.; Stott Reynolds, T. J.; Schehr, R.; Bandari, R. P.; Diebolder, P. J.; Krieger, S.; Xu, J.; Miao, Y.; Rogers, B. E.; Smith, C. J. Matched-pair, (86)Y/(90)Y-labeled, bivalent RGD/bombesin antagonist, [RGD-Glu-[DO3A]-6-Ahx-RM2], as a potential theranostic agent for prostate cancer. *Nucl Med Biol* **2018**, *62-63*, 71-77. DOI: 10.1016/j.nucmedbio.2018.06.001.
(4) Mitran, B.; Varasteh, Z.; Selvaraju, R. K.; Lindeberg, G.; Sörensen, J.; Larhed, M.; Tolmachev, V.; Rosenström, U.; Orlova, A. Selection of optimal chelator improves the contrast of GRPR imaging using bombesin analogue RM26. *Int J Oncol* **2016**, *48* (5), 2124-2134. DOI: 10.3892/ijo.2016.3429.
(5) Popp, I.; Del Pozzo, L.; Waser, B.; Reubi, J. C.; Meyer, P. T.; Maecke, H. R.; Gourni, E. Approaches to improve metabolic stability of a statine-based GRP receptor antagonist. *Nucl Med Biol* **2017**, *45*, 22-29. DOI: 10.1016/j.nucmedbio.2016.11.004.
(6) Abouzayed, A.; Kanellopoulos, P.; Gorislav, A.; Tolmachev, V.; Maina, T.; Nock, B. A.; Orlova, A. Preclinical Characterization of a Stabilized Gastrin-Releasing Peptide Receptor Antagonist for Targeted Cancer Theranostics. *Biomolecules* **2023**, *13* (7). DOI: 10.3390/biom13071134.
(7) Chatalic, K. L.; Konijnenberg, M.; Nonnekens, J.; de Blois, E.; Hoeben, S.; de Ridder, C.; Brunel, L.; Fehrentz, J. A.; Martinez, J.; van Gent, D. C.; et al. In Vivo Stabilization of a Gastrin-Releasing Peptide Receptor Antagonist Enhances PET Imaging and Radionuclide Therapy of Prostate Cancer in Preclinical Studies. *Theranostics* **2016**, *6* (1), 104-117. DOI: 10.7150/thno.13580.
(8) Kanellopoulos, P.; Mattsson, A.; Abouzayed, A.; Obeid, K.; Nock, B. A.; Tolmachev, V.; Maina, T.; Orlova, A. Preclinical evaluation of new GRPR-antagonists with improved metabolic stability for radiotheranostic use in oncology. *EJNMMI Radiopharm Chem* **2024**, *9* (1), 13. DOI: 10.1186/s41181-024-00242-6.
(9) Günther, T.; Deiser, S.; Felber, V.; Beck, R.; Wester, H. J. Substitution of l-Tryptophan by α-Methyl-l-Tryptophan in (177)Lu-RM2 Results in (177)Lu-AMTG, a High-Affinity Gastrin-Releasing Peptide Receptor Ligand with Improved In Vivo Stability. *J Nucl Med* **2022**, *63* (9), 1364-1370. DOI: 10.2967/jnumed.121.263323.
(10) Felber, V.; Holzleitner, N.; Joksch, M.; Suhrbier, T.; von Amsberg, G.; Schwarzenböck, S.; Kurth, J.; Heuschkel, M.; Günther, T.; Krause, B. J. First-in-Human Serum Stability Studies of [(177)Lu]Lu-AMTG: A Step Toward Improved GRPR-Targeted Radiopharmaceutical Therapy. *J Nucl Med* **2025**, *66* (6), 896-899. DOI: 10.2967/jnumed.124.269132.
